# Physicochemical and Mechanical Evaluation of Nanocomposite Cryogels Based on Chitosan and Laponite and Their Potential for Drug Delivery

**DOI:** 10.3390/gels12090771

**Published:** 2026-08-27

**Authors:** Ecaterina Stela Dragan, Maria Valentina Dinu, Maria Marinela Lazar, Daniela Rusu

**Affiliations:** Department of Functional Polymers, “Petru Poni” Institute of Macromolecular Chemistry, Grigore Ghica Voda Alley 41 A, 700487 Iasi, Romania; vdinu@icmpp.ro (M.V.D.); maria.lazar@icmpp.ro (M.M.L.); rusu.daniela@icmpp.ro (D.R.)

**Keywords:** chitosan, laponite RD, cryogels, 5-fluorouracil, drug delivery

## Abstract

It is known that the controlled release of anticancer drugs with low molecular weight from drug delivery systems (DDSs) based on polysaccharides such as chitosan (CS) still encounter certain difficulties because of the high hydrophilicity of the carrier. The release rate is very fast, with a burst release that is usually obvious. Therefore, finding novel systems to carry and release low molecular weight drugs such as 5-fluorouracil (5-FU) is still of interest. The impact of chitosan (CS) characteristics (deacetylation degree and molar mass), and concentration as well as of the strategy employed to fabricate porous nanocomposites CS–laponite (LAP), on the physicochemical properties of CS-LAP sponges was first investigated in the paper. Information about the incorporation of LAP within the CS-LAP nanocomposites was obtained by EDX and FTIR spectroscopy. The influence of the fabrication conditions on the physical cross-linking was evidenced first by the swelling of the composite sponges at equilibrium in distilled water, with lower values of the swelling ratio found for the composites prepared with one freeze-drying (1FD) step, than of those prepared with 2FD steps. The elastic modulus ranged between 6 and 21 kPa for CS-LAP composites prepared with 1FD, and between 2 and 10 kPa for the nanocomposites prepared with 2FD steps. The physicochemical properties of CS-LAP nanocomposite sponges were correlated with their behavior in the loading and in vitro release of 5-FU as a function of pH. It was found that the CS-LAP composites with the highest swelling ratio exhibited the fastest release of 5-FU in simulated gastric fluid (pH 2.0), with the cumulative release in the range of 85–100%, in about 60 min, at 37 °C. The CS-LAP composites with the lowest swelling ratio, displayed the slowest release rate of drug. The drug release from the double component composites was accompanied by the carrier dissolution/disintegration. To retard the release of 5-FU in the gastric environment, the drug was sealed in the porous CS-LAP nanocomposites by simple complexation of the free positive charges of CS with carboxymethylcellulose as polyanion. These systems kept their integrity at pH 2.0, pH 6.8 and phosphate buffer with pH 7.4, and released 5-FU in a controlled manner, making them suitable systems for delivery of 5-FU in both the stomach and small intestine.

## 1. Introduction

The fast evolution of controlled drug delivery systems (DDSs) is motivated by the stringent requirement to enhance the drug selectivity and efficiency, minimizing the high toxicity of anticancer drugs while increasing the clinical efficiency, the drug stability and the patient compliance [1,2,3,4,5,6,7]. Numerous DDSs are based on hydrogels, which are able to absorb and retain large volumes of water without dissolving owing to the physical or chemical cross-links [1,3,5]. Intelligent hydrogels (smart responsive) change their volume in response to a slight variation in the external/internal stimuli, such as pH, temperature, ionic strength, solvent composition, light, electric or magnetic field. These fascinating properties made them especially attractive for biomedical applications such as DDSs, tissue regeneration, wound management, artificial organs, cartilages, and actuators [1,4,5]. Hydrophilic synthetic polymers [8,9,10] and polymers with origins in renewable resources such as polysaccharides [4,11,12,13,14,15,16,17,18,19,20] represent main elements in the construction of biocompatible and biodegradable hydrogels as drug carriers. Among polysaccharides, chitosan (CS), with its biocompatibility, nontoxicity, and intrinsic antibacterial properties, has attracted a huge interest for fabrication of novel drug carriers of various medicines provided with delayed drug release [12,15,21,22,23,24,25]. Polyelectrolyte complexes (PECs) formed from CS and carboxymethylcellulose (CMC) or poly(2-acrylamido-2-methylpropanesulfonate sodium salt) as sponges with tunable structures, morphologies, and mechanical properties were fabricated and their performances in sustained delivery of curcumin (CCM) as an anti-inflammatory drug have been demonstrated [12]. Ulah et al. have formulated folic acid-modified CS nanoparticles by the ionic gelation method and used them as a carrier for 5-FU for colon cancer [15]. Interpenetrating polymer networks (IPNs) of CS and poly(2-hydroxyethyl methacrylate-*co*-itaconic acid) were synthesized [24]. Swelling properties have been evaluated at different pH levels, and found they depended on the CS and itaconic acid content. Controlled release of diclofenac sodium from the prepared composites has been evaluated. IPN hydrogel films based on CS, 2-hydroxyethyl methacrylate and 2-hydroxypropyl methacrylate have been reported by Bayramoglu et al. and their potential for delivery DOX and CCM at pH 5.5 and 7.4 and wound dressing has been investigated [25]. In vitro drug release profiles revealed a time-dependent release pattern, with a maximum release observed at 48 h for all formulations. It was found that a formulation containing CS and HEMA in a 1:1 ratio showed the highest drug release rates with 76.0% for DOX and 75.5% for CCM [25].

The main weak points of hydrogels, consisting of the poor mechanical strength and slow response to stimuli, have been alleviated by diverse strategies including IPNs [2,8,9,24,25], introducing hydrophobic substituents, or engineering hydrogel–silicate nanocomposites [11,13,14,16]. Laponite is a representative silicate that is efficient as a physical cross-linker in the fabrication of nanocomposite hydrogels for biomedical purposes [13,14,16,17,18,19,26], including cell adhesion [13], bone regeneration [16], and drug delivery [14,17,18,26]. Thus, nanocomposite hydrogels have been produced from LAP and polyethylene-glycol diacrylate (PEGDA), with or without Irgacure (IG), for application in bone tissue regeneration. The FTIR results indicated that PEGDA polymer chains were entangled between the LAP dis in the composite hydrogel. It has been evidenced that these nanocomposite hydrogels would have potential for bone regeneration [16]. Luo et al. have recently reported fabrication of natural polymer composite gels with sensitivity to near-infrared (NIR) light through free radical polymerization of vinyl-functionalized dextran (DexIEM), vinyl-modified graphene oxide (GM), and LAP [17]. With the increase in LAP content, the density of cross-linking in the hydrogel increased, and its mechanical properties have been improved. Under NIR irradiation, the DexIEM-GM-LAP hydrogel exhibited a photothermal property, where the surface temperature increased from 26.8 to 55.5 °C. The results of the antibacterial performance tests after loading with ciprofloxacin indicated remarkable antibacterial effect. Various CS-LAP nanocomposites have been designed [26,27,28,29,30,31,32,33,34,35,36,37,38] either as injectable hydrogels [30,33], carriers for anticancer drugs [21,31,32,34,35], antibiotics [27,36], or cell proliferation for wound healing [38]. Series of CS/LAP composite beads have been prepared by ionic cross-linking with TPP as the ionic cross-linker to test as DDS for Ofloxacin as model drug [21]. The swelling behavior in physiological pH solutions (7.4 and 1.2), drug encapsulation efficiency and controlled release behavior have indicated that the incorporation of LAP remarkably improved the swelling behavior, enhanced the drug entrapment efficiency, and slowed down the drug release. Ordikhani et al. have prepared CS–LAP nanocomposite coatings by an electrophoretic deposition technique on the surface of titanium foils with bone regenerative potential and controlled drug release capacity [27]. By applying an electric field, the charged particles were deposited on the surface of titanium foils and uniform chitosan films containing 25–55 wt.% LAP and vancomycin have been obtained. A pH-responsive drug carrier based on CS and LAP RD was developed for the controllable release of ofloxacin in the gastric environment [29]. Hydroxyapatite (HA) was grafted into CS/LAP matrix to overcome the burst release of the CS/LAP. Heragh et al. have investigated the co-delivery of DOX and Saffron extract (SAF) from cross-linked CS/LAP RD nanoparticles with the final purpose of reducing the side effects of DOX and enhancing the effectiveness of drugs [31]. The results indicated that the release of DOX and SAF remarkably increased by decreasing pH from neutral (pH 7.4) to acidic (pH 5.5). An increase in cross-linked CS content contributed to a sustained release behavior by decreasing the release rate. By the combination of magnetic LAP RD nanodiscs and CS/κ-carrageenan (Car) biopolymers, Mahdavinia et al. have prepared core–shell nanocarriers with excellent pH-responsiveness to load and release DOX as an anticancer drug model [32]. A sustained release of DOX was observed, which suggests the core–shell nanocarriers as promising candidates for DDSs. In another approach, LAP nanodiscs have been loaded with the antibacterial drug kanamycin (KANA) before being embedded into a poly(lactic-*co*-glycolic acid) (PLGA) membrane and coated with the biopolymer CS [36]. Results indicated that these biocompatible materials combined the excellent capacity of LAP for controlled drug release with the mechanical robustness of PLGA and the antibacterial properties of CS as well as its hydrophilicity to form a composite highly suitable as an infection-preventing wound dressing. The composite hydrogel PLGA/LAP/KANA/CS released drugs in vitro in a sustainable manner over 30 days, which completely inhibited the growth of infectious bacteria, prompted the adhesion of fibroblasts, and accelerated their proliferation.

5-Fluorouracil (5-FU) is one of the most recommended chemotherapeutic drugs in the treatment of tumors including gastric, colorectal, breast, liver, skin and lung but it suffers from low bioavailability, short half-time in the blood circuit system and high toxicity [39,40,41,42]. Moreover, the lack of drug specificity damages the healthy tissues beside the targeted cancer cells [39]. Therefore, there is a stringent need to find novel carriers endowed with as smart as possible control of the release of 5-FU, with high cell selectivity, enhanced stability and bioavailability of the encapsulated drug. In this context, pH-responsive carbohydrate polymers have gained significant attention as promising candidates for the next generation of oral DDSs. The gastrointestinal tract (GIT), with its distinct pH environments, constitutes a suitable platform for the investigation of performances of pH-controlled DDSs [2,4,41]. Various biodegradable composite hydrogels based on CS as chemically cross-linked films [40], ionically cross-linked nanoparticles [41], m-PEG-CS nanogels [42], alginate-CS/montmorillonite nanocomposites [43], CS microspheres [44,45], electrospun nanofibrous composite mats based on CS and polyvinylpyrrolidone containing graphene oxide [46], suitable for oral delivery of 5-FU with the main purpose of colorectal cancer therapy, have been recently designed. In another approach, anticancer 5-FU drug was loaded into CS/polyacrylic acid/Fe_3_O_4_ magnetic nanocomposite hydrogel through immersing the nanocomposite in the drug solution [47]. It was demonstrated that the porous CS-based nanocomposites can hold large amounts of drugs within their network, with controlled and sustained release of drugs while ensuring an enhanced stability of the payload [39]. Coating the drug carrier with synthetic or natural polymer-bearing carboxylic groups can provide the opportunity to deliver drugs directly to the large bowel because these polymers can prevent drug release and degradation in the acidic environment of the stomach [42,48,49,50]. Thus, 5-FU has been encapsulated into the porous Zn-based metal–organic framework (MOF-5) and CMC has been used to cover 5-FU@MOF-5 hybrid and to protect the drug through the digestive system [48]. However, it was found that the CS-LAP nanocomposite sponges have not been evaluated as regards the potential carrier and pH-controlled release of 5-FU.

Therefore, the main objective of the present study was to investigate first the impact of the swelling rate and morphology (the average pore size, and pore size distribution) of the CS-LAP composite sponges, tailored to the CS characteristics, the concentration of CS, as well as the strategy employed to generate porous nanocomposite materials, on the delivery profile of 5-FU controlled by pH. It was also postulated that by the free positive charges of CS, which can be complexed with a polyanion such as CMC, after the drug loading, these nanocomposites will offer a plentiful platform for a systematic investigation of the opportunities to release 5-FU in response to the specific pH of the three compartments of GIT.

## 2. Results and Discussion

In this work, a series of 3D and porous nanocomposite cryogels were developed from CS and LAP to be evaluated for their potential in controlled delivery of 5-FU. The fabrication of CS-LAP nanocomposite sponges and their performances in wound healing have been recently reported by the authors [38]. A constant concentration (4 wt.%) of CS with molar mass of 207 kDa and deacetylation degree (DD) of 94%, with LAP concentrations varied in the range 0.2–2 wt.%, have been previously used. To optimize the properties of CS-LAP macroporous nanocomposites, making them suitable as DDSs, the molar mass and the DD of CS, and the concentration of CS and LAP in the initial mixture of components were systematically varied in this work, as presented in Table 1.

**Scheme 1:**
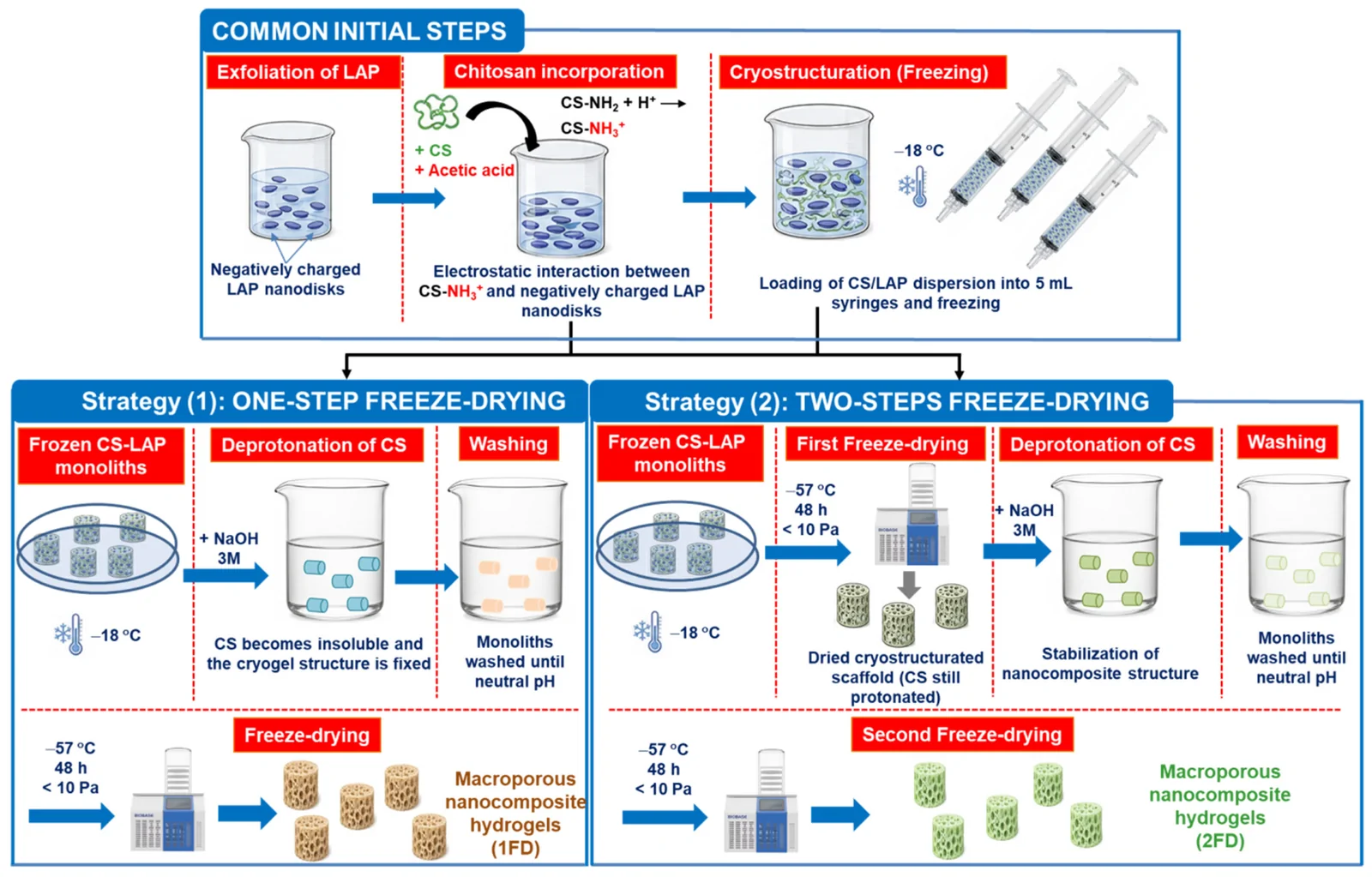
Main steps involved in the preparation of CS-LAP sponges according to the two strategies developed in this work: strategy (1) consisting of one-step freeze-drying; strategy (2) consisting of two-step freeze-dryings.

Our interest in the tight control of the nanocomposite porosity conducted to the fabrication of CS-LAP sponges through two approaches, as presented in Scheme 1: (1) cryostructuration in one step, when the homogeneous dispersion of LAP nanodisks in aqueous solution of protonated CS was first frozen and then the stabilization of the nanocomposite cryogel morphology was achieved by the deprotonation of CS in NaOH 3 M, followed by washing at neutral pH and then freeze-drying, coded with 1FD; (2) cryostructuration of the CS-LAP nanocomposites in two steps, achieved by freeze-drying first the frozen dispersion of LAP in aqueous solution of protonated CS, followed by the deprotonation of CS in NaOH 3 M, washing at neutral pH, and again freeze-drying to reach stable macroporous nanocomposites, coded with 2FD.

As can be seen in Scheme 1, after the exfoliation of LAP in H_2_O, the CS particles were spread between the LAP nanodisks with the final formation of a colloidal dispersion of CS and LAP. By the addition of acetic acid, the formed protonated CS chains (CS-NH_3_^+^) come in contact with the negatively charged surface of LAP disks. We are expecting the LAP disk distribution to be influenced by the molar mass and DD of CS, CS concentration and the ratio between CS and LAP in the initial mixture. Both series of composite sponges underwent comprehensive characterization to decide the optimum conditions for the fabrication of CS-LAP nanocomposites as potential DDSs for 5-FU.

### 2.1. Characterization of CS-LAP Nanocomposites

The influence of the synthesis conditions on the formation yield of the CS-LAP sponges was not so dramatic, as the values of yield in Table 1 show. As a general trend, at concentration of CS of 3 wt.%, the yield values, evaluated with Equation (4), were comparable, irrespective of the synthesis strategy (1FD or 2FD) and CS characteristics, i.e., around 87–89%. However, the highest values of the formation yield were found in the case of the composites prepared with CS4 (the highest DD, 94%), with 91.86% and 89.13%, for 1FD and 2FD, respectively, suggesting that the cross-linking efficiency was the most effective when the CS with the highest content of cationic centers was involved in the sponge formation. The yield in nanocomposite sponges was the lowest when the concentration of CS in the initial mixture of CS and LAP was only 1 wt.% (81.56%, for the sample CS3.1-LAP0.1.2FD). The above results support the opportunity to modulate the CS-LAP nanocomposite properties by the CS characteristics, CS concentration and CS:LAP ratio.

#### 2.1.1. Internal Morphology and Pore Size Distribution

**Figure 1:**
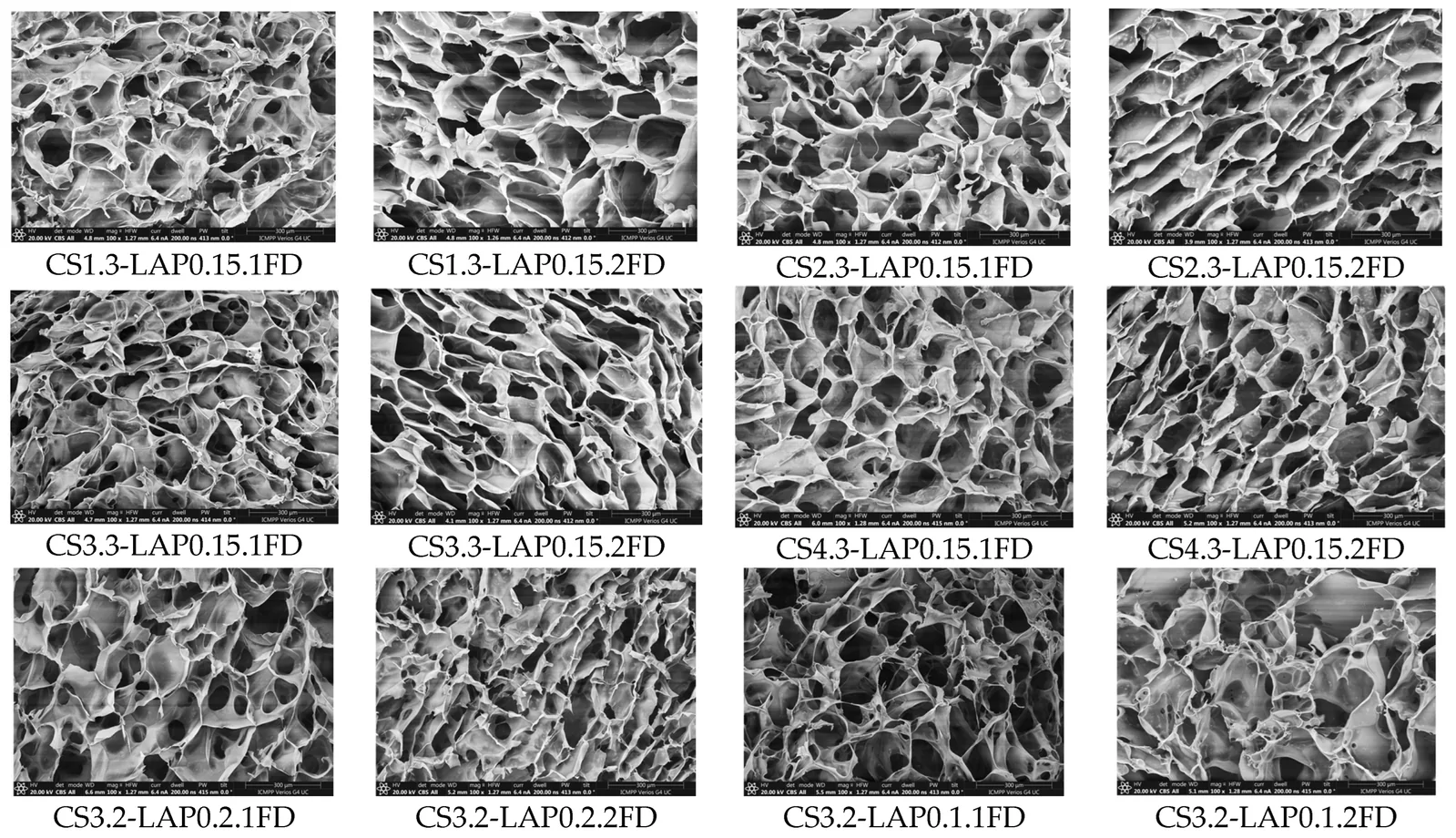
SEM micrographs of the CS-LAP nanocomposites as a function of synthesis conditions. Scale bar was 300 µm and mag 100× in all micrographs. Sample code is as presented in Table 1.

It was expected for the intrinsic characteristics of CS (molar mass and DD), the CS concentration, the CS:LAP ratio as well as the engineering of the composite porosity to behave as instruments to tailor the composite morphology, as shown for other porous materials [51,52,53]. Figure 1 presents the SEM micrographs of nanocomposites at the same magnification (100×).

At first sight, the composites prepared by the 2FD strategy display much more regular pores, for all samples prepared with CS concentration of 3 wt.% (CS1.3-LAP0.15.2FD, CS2.3-LAP0.15.2FD, CS3.3-LAP0.15.2FD and CS4.3-LAP0.15.2FD) compared with those prepared by the 1FD strategy (CS1.3-LAP0.15.1FD, CS2.3-LAP0.15.1FD, CS3.3-LAP0.15.1FD and CS4.3-LAP0.15.1FD).

Decreasing the concentration of CS from 3 wt.% to 2 wt.% (CS3.2-LAP0.2.1FD, CS3.2-LAP0.2.2FD, CS3.2-LAP0.1.1FD and CS3.2-LAP0.1.2FD) contributed to the diminishing of the differences in sponges morphology.

**Figure 2:**
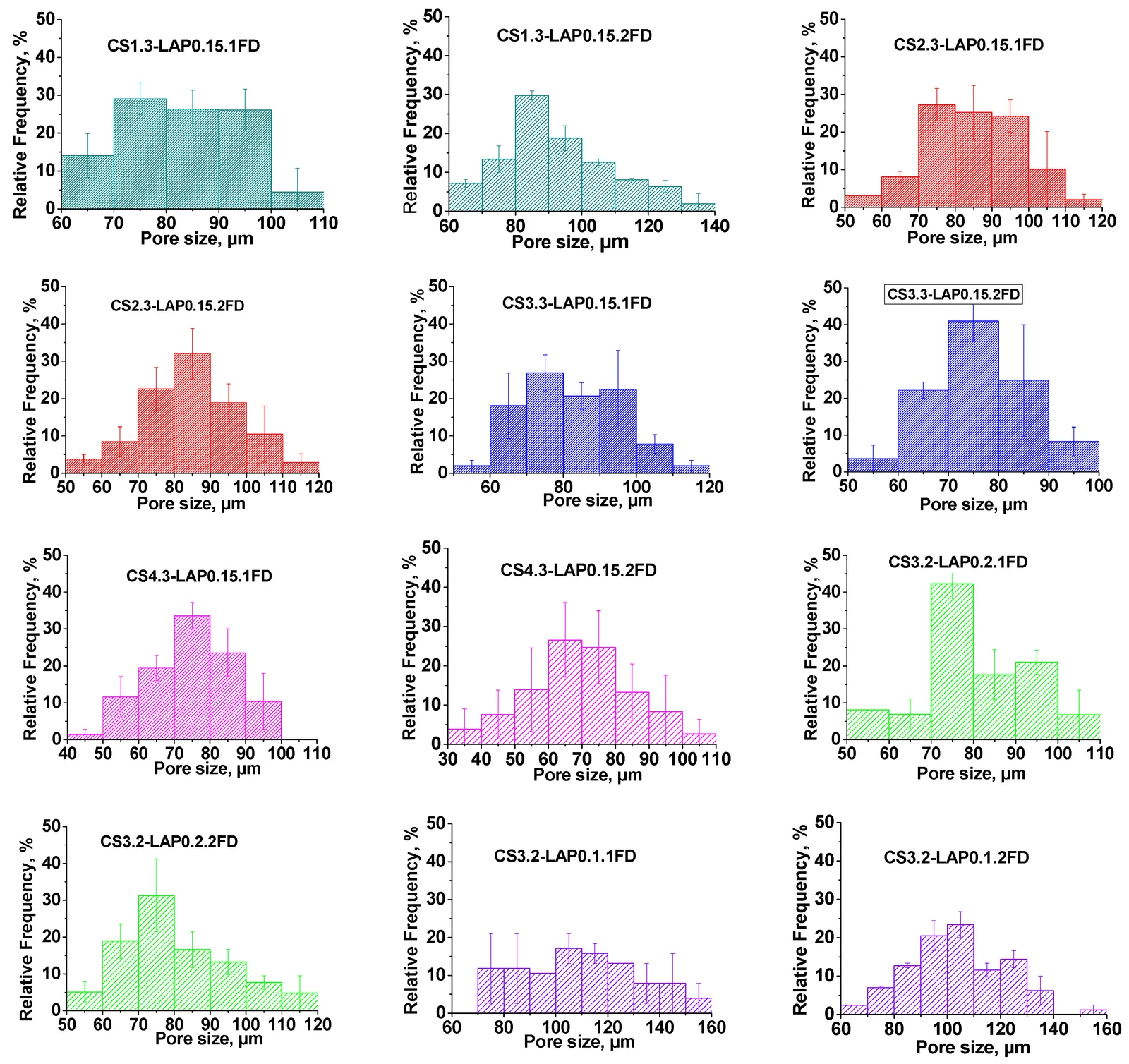
Histograms of the pore size distribution of the CS-LAP nanocomposite sponges based on different CS samples, CS concentration and CS:LAP ratio and synthesis strategy.

The pore size distribution presented in Figure 2 indicates a relative narrow polydispersity for all composites prepared at CS concentration of 3 wt.%, with a relative frequency of ~90% for pores with sizes in the range 60–100 µm. However, the pore size distribution is even narrower in the case of the nanocomposites prepared by the strategy 2FD (CS1.3-LAP0.15.2FD, CS2.3-LAP0.15.2FD, CS3.3-LAP0.15.2FD), excepting the nanocomposite prepared with CS4, where the pore size distribution is narrower in the case of the composite prepared by the strategy 1FD. The decrease in the CS concentration at 2 wt.% contributed to the increase in the pore size polydispersity, more in the case of the lower content of LAP (CS3.2-LAP0.1.1FD and CS3.2-LAP0.1.2FD), with the largest pores found in the case of these composite sponges. These results clearly support the possible correlations between the CS characteristics, CS concentration and CS:LAP ratio in the forecast of the nanocomposite morphology and pore size distribution. The narrow distribution of pore sizes found in the case of the nanocomposite sponges mentioned above would be helpful in predicting the sustained drug release profiles.

To show the presence and the distribution of LAP nanodisks in the CS-LAP sponges, SEM micrographs at a magnitude of 20,000× were included in the Supplementary Materials (Figure S1). As can be seen, the LAP nanodisks are almost evenly distributed into the nanocomposite walls. The presence of a higher amount of LAP nanodisks is evident in the SEM micrograph of the CS3.2-LAP0.2 nanocomposites, where the CS:LAP ratio was 10:1, compared with CS3.2-LAP0.1, with CS:LAP ratio of 20:1.

**Figure 3:**
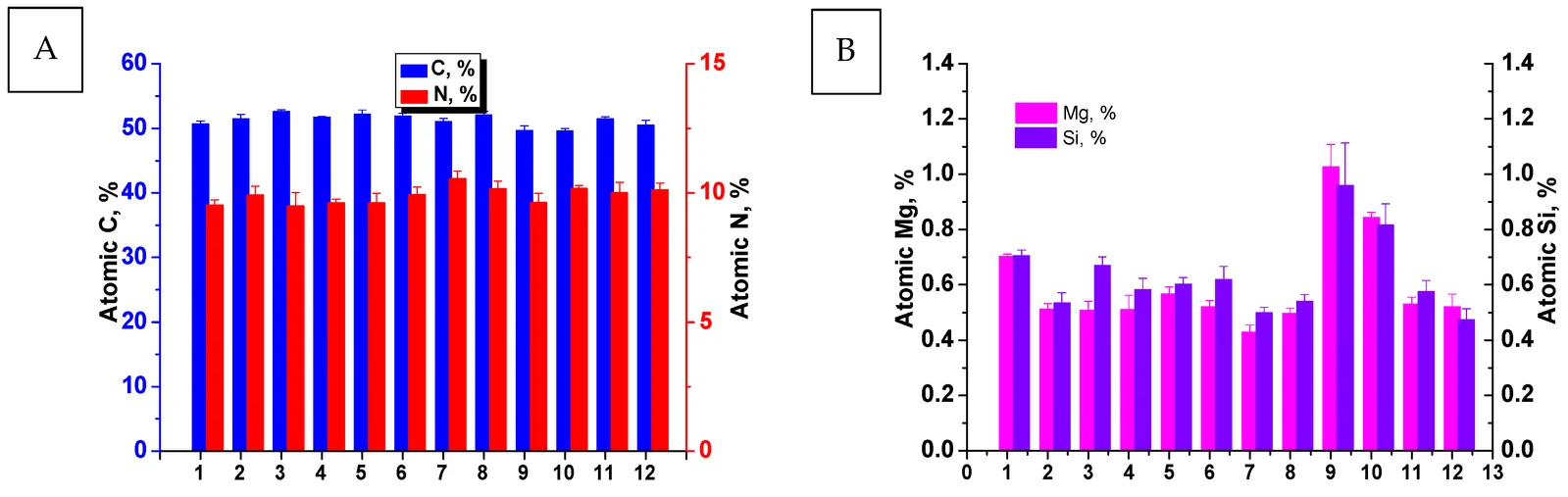
EDX data of C and N (**A**), Mg and Si (**B**) for the nanocomposite sponges CS-LAP: (1) CS1.3-LAP0.15.1FD; (2) CS1.3-LAP0.15.2FD; (3) CS2.3-LAP0.15.1FD; (4) CS2.3-LAP0.15.2FD; (5) CS3.3-LAP0.15.1FD; (6) CS3.3-LAP0.15.2FD; (7) CS4.3-LAP0.15.1FD; (8) CS4.3-LAP0.15.2FD; (9) CS3.2-LAP0.2.1FD; (10) CS3.2-LAP0.2.2FD; (11) CS3.2-LAP0.1.1FD; (12) CS3.2-LAP0.1.2FD.

The EDX spectra of the CS-LAP nanocomposite sponges are presented in Figure S2 and the element content as Atomic % (C, N, Mg and Si), as a function of the synthesis conditions of nanocomposites, can be seen in Figure 3. The EDX data unambiguously demonstrate the incorporation of LAP nanoparticles within the nanocomposite sponges at a level well correlated with the synthesis conditions. Thus, at a CS concentration of 3 wt.% and a ratio CS:LAP of 20:1, the content in atomic C was in the range of 49.59–52.65%, and that of atomic N was in the range of 9.49–10.55% (Figure 3A), while the values of Mg and Si were in the range of 0.43–1.03%, and 0.475–0.96% (Figure 3B), respectively, irrespective of the synthesis strategy. Increasing the content of LAP in the initial mixture of CS and LAP (CS:LAP ratio of 10:1) conducted to the decrease in the atomic C at 49.52 and 49.50% for CS3.2-LAP0.2.1FD and CS3.2-LAP0.2.2FD, respectively (Figure 3A), and to the increase in atomic Mg at 1.0275 ± 0.08% for CS3.2-LAP0.2.1FD, and 0.8425 ± 0.0192%, for CS3.2-LAP0.2.2FD, and in Si at 0.96 ± 0.152% for CS3.2-LAP0.2.1FD and 0.817 ± 0.0746%, for CS3.2-LAP0.2.2FD (Figure 3B) compared with about half of these values found for CS3.2-LAP0.1.1FD and CS3.2-LAP0.1.2FD composites, different only by the CS:LAP ratio (20:1).

The EDX percentages of the atomic O and Na, for all CS-LAP composites, are presented in Figure S3.

#### 2.1.2. Swelling Kinetics of CS-LAP Nanocomposite Sponges

**Figure 4:**
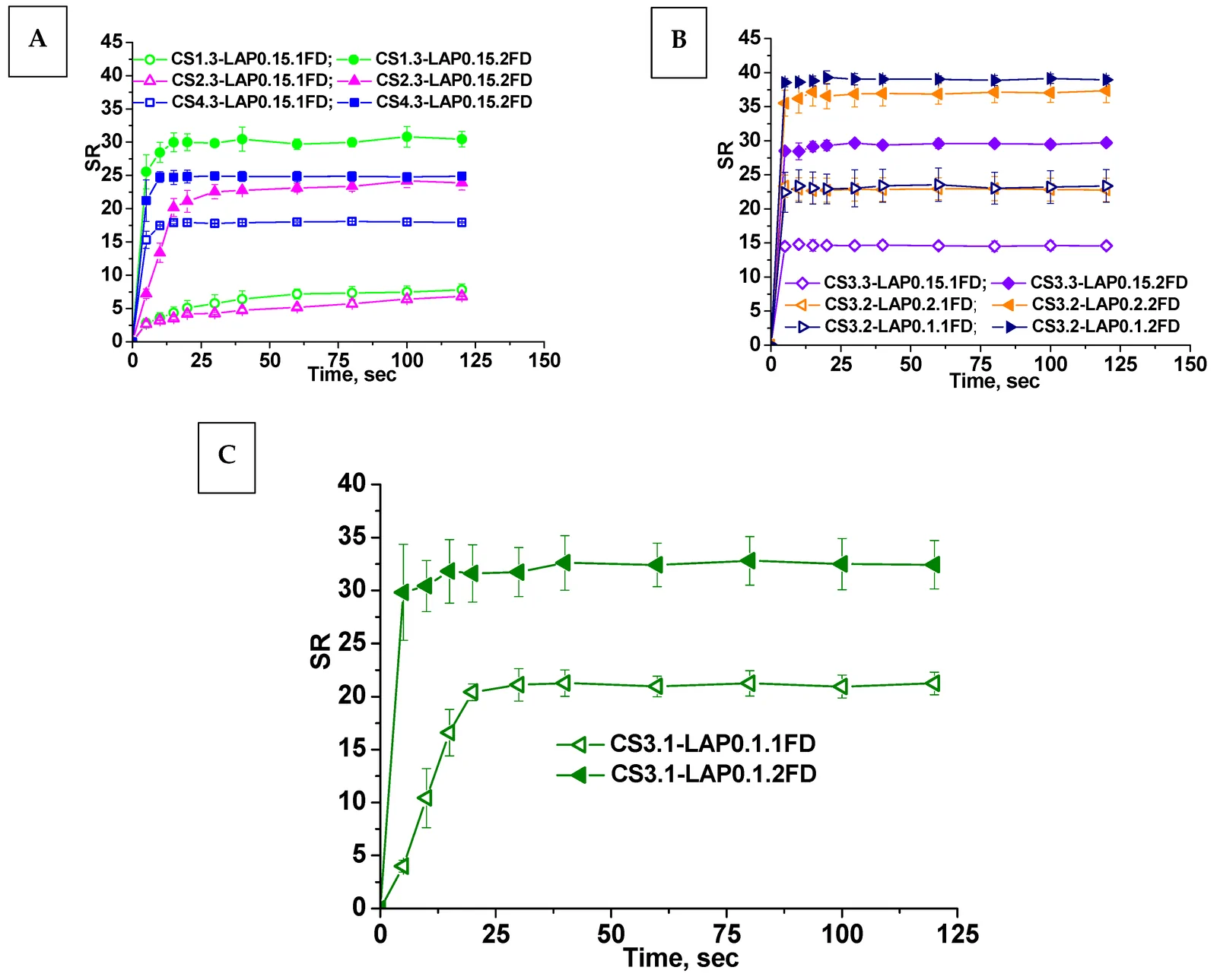
Swelling kinetics of the CS-LAP composites as a function of CS molar mass and DD (**A**), CS:LAP ratio (**B**) and CS concentration (**B**,**C**).

The values of the swelling ratio (SR), calculated with Equation (5) and plotted in Figure 4 as a function of time, show that a common characteristic of the CS-LAP composites is their very fast swelling (with the equilibrium of swelling reached in 5–10 s), a behavior which supports the strong connectivity of their pores [8,12,53,54].

The influence of CS molar mass and DD on the swelling kinetics, tightly correlated with the level of physical interactions in these nanocomposites, can be seen in Figure 4A.

As can be observed, the SR values of the composites CS1.3-LAP0.15 and CS2.3-LAP0.15, constructed with CS and characterized by the same DD but with different molar masses (Table 1), are comparable in the case of composites with CS deprotonation just after freezing (SR = 7.79 ± 0.87 and 6.84 ± 0.05, for CS1.3-LAP0.15.1FD and CS2.3-LAP0.15.1FD, respectively). The values of SR were much higher in the case of composites prepared with 2FD steps. This shows that the deprotonation of CS before freeze-drying contributed to a more compact morphology compared with that which resulted when the freezing was followed by freeze-drying, with the CS deprotonation after that. A higher value of SR was observed for the composite prepared with CS1 (SR = 30.45 ± 1.16) than that of the composite prepared with CS2 (SR = 23.88 ± 1.07). The SR value of the composite CS1.3-LAP0.15, higher than that of the composite CS2.3-LAP0.15, can be attributed to a higher cross-linking density (a more compact structure) in the case of the sample prepared with the highest molar mass (M_v_ = 305 kDa) than that of the composite prepared with CS1 (M_v_ = 147 kDa). Therefore, to conclude, the influence of CS molar mass could be revealed only by the comparison of the samples obtained with 2FD steps, with a higher molar mass favorable for a greater number of physical cross-links.

Information about the influence of DD of CS on the swelling kinetics could be found by the comparison of the SR values obtained for CS1.3-LAP0.15 (M_v_ = 147 kDa and DD = 82%) with those obtained for the composite CS4.3-LAP0.15 (CS with M_v_ = 207 kDa and DD = 94%, Figure 4A). Thus, the SR values of the composites CS1.3-LAP0.15.1FD and CS4.3-LAP0.15.1FD were 7.79 ± 0.87 and 17.95 ± 0.1, respectively, while those of the composites CS1.3-LAP0.15.2FD and CS4.3-LAP0.15.2FD were 30.34 ± 1.16 and 23.89 ± 0.57, respectively. As can be seen, the influence of DD on the efficiency of physical cross-linking was much stronger than that of the CS molar mass. It is evident that the composite CS4.3-LAP0.15.1FD was less compact than the composite CS1.3-LAP0.15.1FD, while in the case of composites prepared with 2FD steps the order was reversed (CS4.3-LAP0.15.2FD more compact than CS1.3-LAP0.15.2FD). The values of SR for the nanocomposites CS4.3-LAP0.15.1FD and CS4.3-LAP0.15.2FD are in agreement with the similar pore size distribution found for these samples (Figure 2).

CS concentration was another parameter of interest for the optimization of the CS-LAP nanocomposite properties. The SR values vs. time for the composites CS3.3-LAP0.15 and CS3.2-LAP0.1, both 1FD and 2FD, differing by the concentration of CS (Table 1), with the ratio CS:LAP of 20:1 in all cases, are plotted in Figure 4B. The values of SR were: 14.59 ± 0.56 and 29.17 ± 0.58 for CS3.3-LAP0.15.1FD and CS3.3-LAP0.15.2FD, respectively, and 23.36 ± 2.4 and 39 ± 0.7 for CS3.2-LAP0.1.1FD and CS3.2-LAP0.1.2FD, respectively. The SR values were much higher for the composites prepared with 2%, *w*/*v* CS, compared with those prepared with 3%, *w*/*v* CS, for both 1FD and 2FD, and this indicates the decrease in the composite compactness with the decrease in CS concentration.

The influence of the CS:LAP ratio was evidenced first in Figure 4B by the comparison of composites CS3.2-LAP0.2.1FD and CS3.2-LAP0.2.2FD, where the CS:LAP was 10:1, with the composites CS3.2-LAP0.1.1FD and CS3.2-LAP0.1.2FD, where the CS:LAP was 20:1. The SR values of composites CS3.2-LAP0.2.1FD and CS3.2-LAP0.2.2FD were 22.78 ± 1.7 and 37.35 ± 1.4, respectively. The influence of the CS:LAP ratio is more consistent for the composites with 2FD, with SR values lower for CS:LAP = 10:1 (37.35 ± 1.4 for CS3.2-LAP0.2.2FD) than for 20:1 (39 ± 0.7 for CS3.2-LAP0.1.2FD). This indicates that a higher density of cross-links was attained at a lower CS:LAP ratio. The SR values for the nanocomposite sponges prepared with CS3 at a concentration of 1 wt.% and CS:LAP ratio of 10:1 are displayed in Figure 4C. The SR values found at equilibrium were 21.25 ± 1.07 for CS3.1-LAP0.1.1FD and 32.4 ± 2.3 for CS3.1-LAP0.1.2FD, values which are lower than those found for CS concentration of 2 wt.%, and the same CS:LAP ratio (Figure 4B).

Swelling was also evaluated in simulated gastric fluid (pH 2.0) and simulated intestinal fluid (pH 6.8) for the formulations which will be involved in the drug release study (Table S2). The formulation CS1.3-LAP0.15.1FD, prepared from CS with a molecular weight of 147 kDa, exhibited relatively low swelling ratios at pH 2 (3.224) and pH 6.8 (3.347), whereas the corresponding 2FD formulation with the same composition showed higher SR (5.570 and 8.878, respectively). This difference suggests that the freeze-drying conditions had an important effect on the internal structure and porosity of the materials. Increasing the CS molecular weight from 147 to 207 kDa while maintaining the CS:LAP ratio at 20:1 resulted in a marked increase in swelling at pH 2, from 5.570 to 9.641. A further increase to 256 kDa, together with a reduction in the CS concentration from 3 to 2% and an increase in LAP concentration from 0.15 to 0.20%, produced the highest swelling ratio at pH 2 (16.701). Generally, the swelling degree was higher at pH 6.8 than at pH 2, particularly for the 2FD formulations. This behavior may be related to the ionization of the CS amino groups and changes in the electrostatic interactions within the CS–LAP network. At pH 2, the samples became unstable, leading to partial or complete disintegration of the polymeric network. Therefore, the lower swelling values observed at pH 2 may be mainly due to sample disintegration. However, the CS3.2-LAP0.2.2FD formulation showed a decrease from 16.701 at pH 2 to 11.274 at pH 6.8, indicating that the swelling response is governed by a combination of molecular weight, CS-LAP composition, and the internal structure generated during synthesis. In conclusion, the results indicate that molecular weight, CS-LAP composition, and freeze-drying conditions together influence the swelling properties of the CS–LAP materials. The 2FD formulations generally exhibited greater swelling than the 1FD formulation, suggesting that processing conditions substantially affected the porous structure of the hydrogels.

#### 2.1.3. Structural Characterization by FTIR Spectroscopy

**Figure 5:**
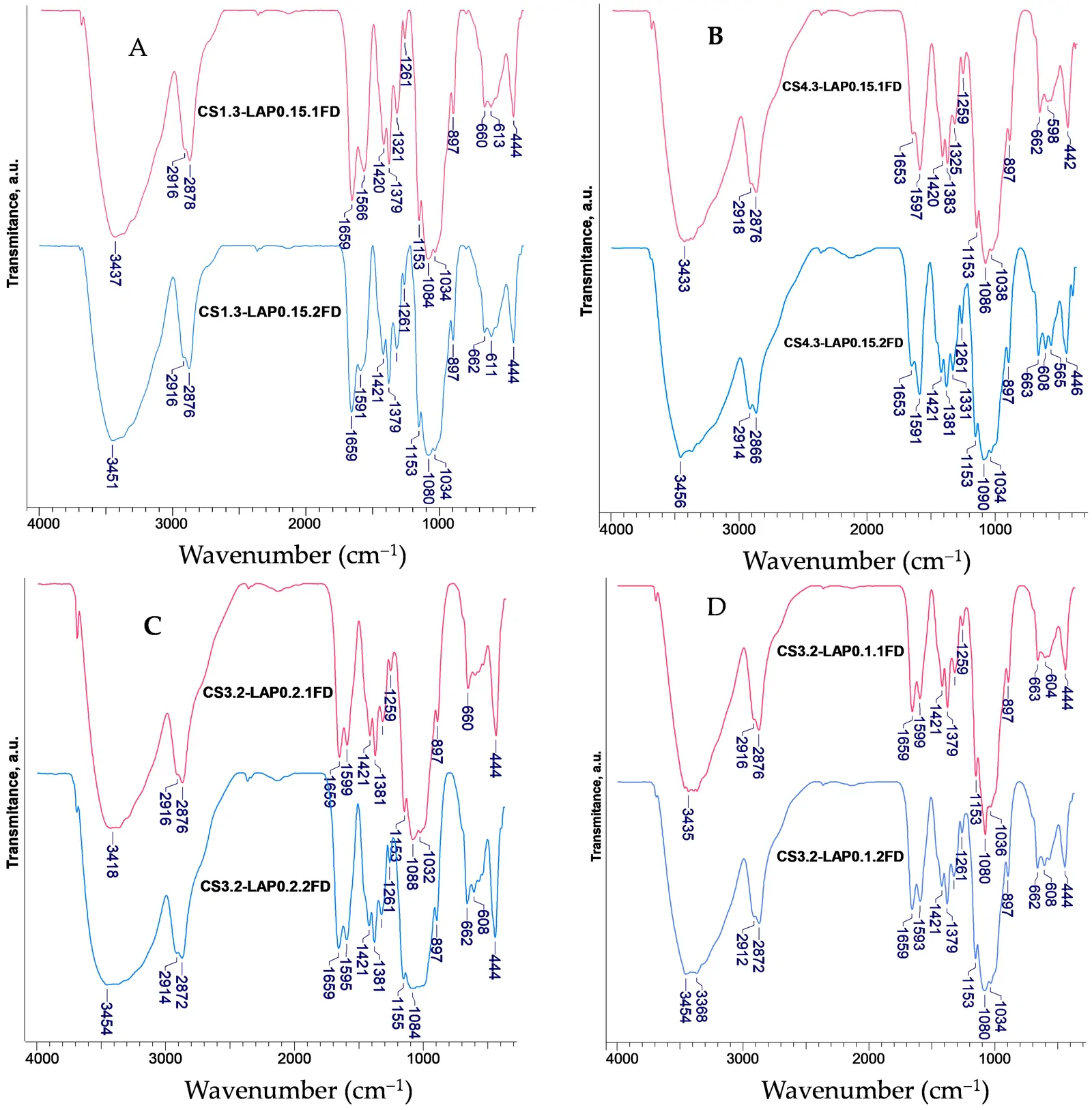
FTIR spectra of CS-LAP nanocomposite sponges prepared with CS1 (**A**), CS4 (**B**) and CS3 at CS concentration of 2 wt.% (**C**,**D**) (the sample code is that of sets presented in Table 1).

The chemical structures of all CS-LAP nanocomposites were evidenced by the FTIR spectroscopy (the FTIR spectra presented in Figure 5 and Figure S4 in the Supplementary Materials), with the FTIR spectra of CS1 and LAP included in Figure S4 for comparison. The characteristic bands of CS, visible in Figure S4A at 1651 cm^−1^, 1599 cm^−1^, 1425 cm^−1^, 1381 cm^−1^, 1321 cm^−1^, and 1261 cm^−1^, were assigned to the C=O stretching vibration in acetamide groups (amide I), NH in primary amino groups, C–H and –CH_2_ stretching [55,56,57], the symmetrical deformation mode of CH_3_ [58], C-N in amide III, and the C-N stretching modes, respectively. The band characteristic to NH vibration in amide (amide II), normally located at 1550 cm^−1^, is missing, possibly overlapped by other bands [56].

The fingerprint bands of CS located at 1157 cm^−1^, ~1070 cm^−1^, and ~1030 cm^−1^ are assigned to the stretching vibrations of the C-O-C bonds in polysaccharides, while the small peak at ~895 cm^−1^ corresponds to the wagging of the saccharide structure of CS. As can be seen in the spectra of CS-LAP nanocomposite sponges and in Table S1, the main bands are located at around 2916, 2878, 1659, ~1590, 1420, 1379, 1321, 1261, 1153, 1084, and 1034 cm^−1^. It is obvious that no consistent changes took place in the shape, location and intensity of the main FTIR bands and this supports that only physical interactions occurred between CS and LAP.

The incorporation of LAP within the nanocomposite is supported by the bands situated at around 660 cm^−1^, associated with Mg-O-Mg bending vibration in LAP; the characteristic band of LAP, located at 465 cm^−1^ in the spectrum of LAP (Figure S4B), and assigned to Si-O-Mg stretching vibration bonds [59], is shifted at around 444 cm^−1^ in all FTIR spectra of nanocomposites and conform the incorporation of LAP within all nanocomposite sponges and the strong electrostatic interaction between CS and LAP.

#### 2.1.4. Elasticity and Shape Memory of CS-LAP Nanocomposites

**Figure 6:**
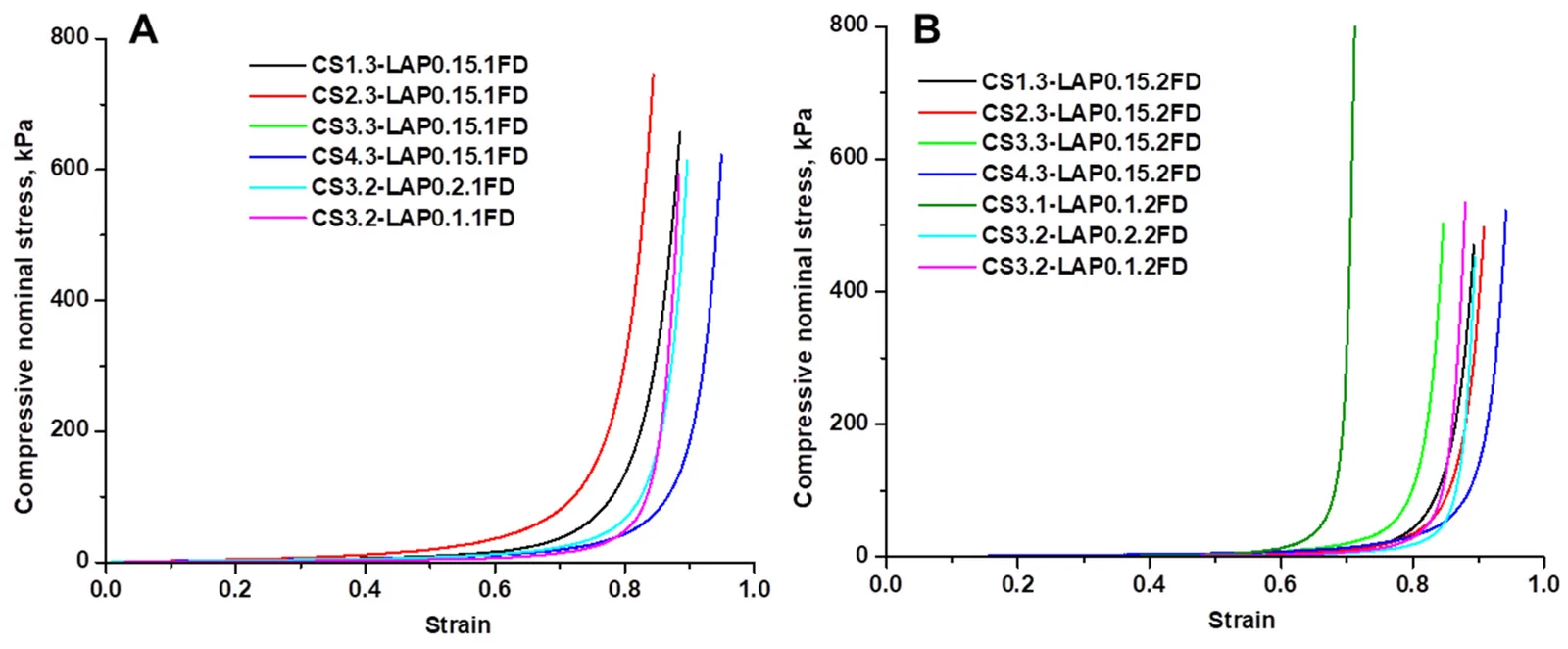
Compressive stress–strain behavior of CS–LAP nanocomposites for (**A**) series “1FD” and (**B**) series “2FD”. The curves correspond to samples with varying CS molar mass, DD, CS concentration, and CS:LAP ratio.

**Figure 7:**
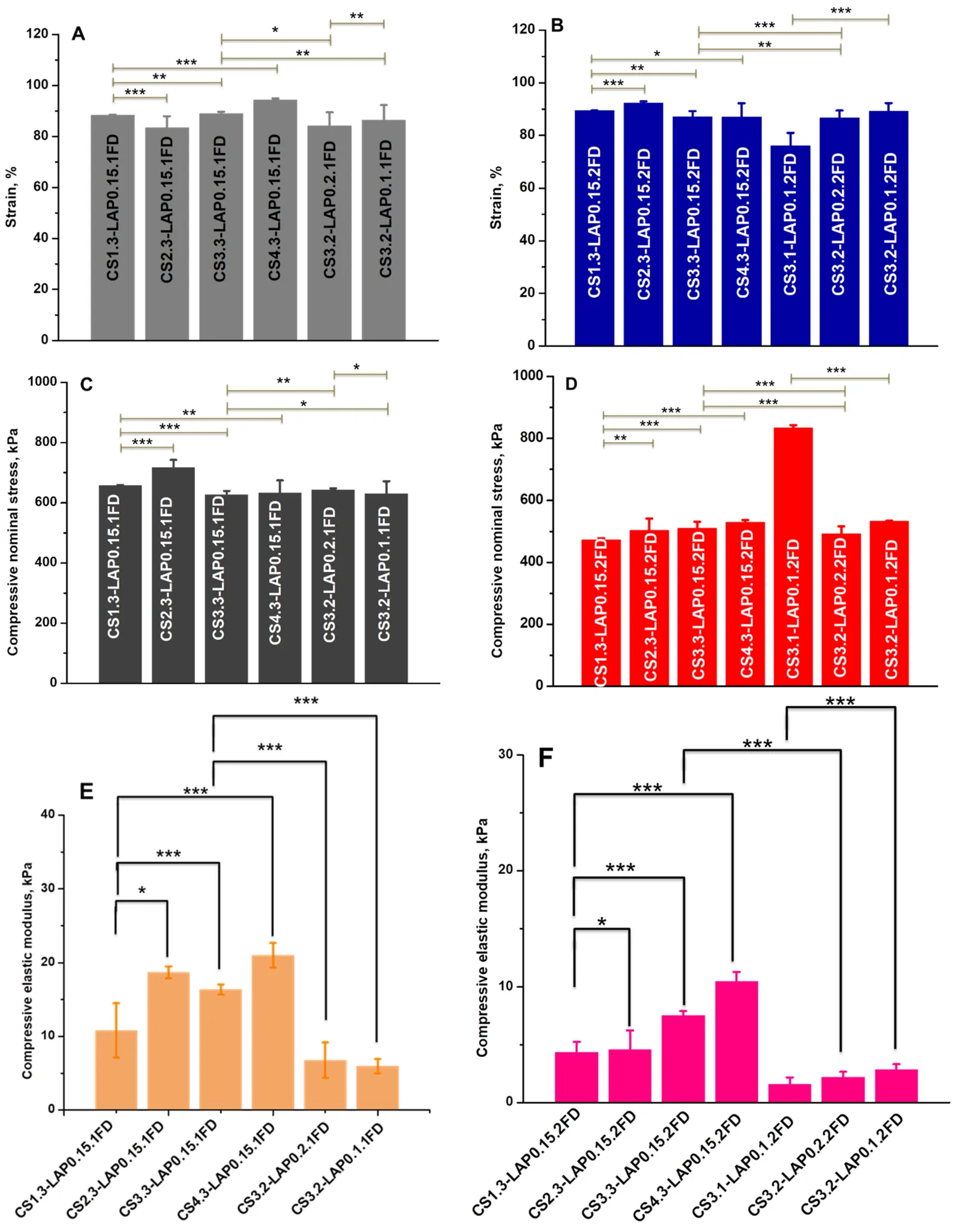
Mechanical properties of CS–LAP nanocomposites for samples “1FD” and “2FD”. (**A**,**B**) strain at maximum compression (%), (**C**,**D**) compressive nominal stress (kPa), and (**E**,**F**) compressive elastic modulus (kPa) for the investigated samples. Results from at least five independent compression experiments are presented as mean ± SD. Statistical significance was assessed using repeated-measures ANOVA and paired-samples *t*-tests performed in JASP. Statistical significance is indicated by * *p* ≤ 0.05, ** *p* ≤ 0.01, and *** *p* ≤ 0.001.

The mechanical performance of the CS–LAP nanocomposites (series “1” and “2”) is governed by the combined effects of CS molar mass, degree of deacetylation, CS concentration, and LAP content, which together determine network organization and reinforcement efficiency (Figure 6 and Figure 7, Tables S3 and S4).

As Figure 6 shows, all samples exhibit a typical nonlinear stress–strain behavior characteristic of polymer–clay nanocomposite hydrogels, with an initial low-stiffness region followed by a sharp stress increase at higher strain.

This strain-hardening behavior is generally associated with progressive network densification and increasing resistance to deformation. Although the stress–strain profiles differ among formulations, all nanocomposite hydrogels maintain high compressibility, with strains ranging from approximately 76 to 94% (Figure 6A,B).

The mechanical parameters obtained from compression testing are summarized in Figure 7 and Figure S5, Tables S3 and S4. The compressive elastic modulus mainly reflects the resistance of the initial porous network to deformation, whereas the stress attained at large strain is additionally governed by pore collapse, contact formation between adjacent pore walls, and progressive densification of the polymer-rich phase. Consequently, the modulus and compressive stress do not necessarily follow the same trend. For the “1” series, increasing the CS molar mass from 147 to 305 kDa (CS1.3-LAP0.15.1FD and CS2.3-LAP0.15.1FD) results in a clear enhancement of mechanical properties, with the compressive elastic modulus rising from 10.82 to 18.68 kPa and the compressive stress from 658.25 to 717.37 kPa (Figure 7, Table S3). This reflects improved chain entanglement and more efficient stress transfer. However, the dependence is not strictly linear, as CS3.3-LAP0.15.1FD (256 kDa) exhibits intermediate modulus values (16.36 kPa) and a lower compressive stress (627.00 kPa), indicating that network organization and intermolecular interactions also contribute to the mechanical response.

The DD has a pronounced effect, with CS4.3-LAP0.15.1FD (94% DD) exhibiting the highest modulus (20.98 kPa), along with high compressive stress (633.07 kPa) and the largest strain (94.40%) (Figure 7, Table S3). The increased rigidity can be attributed to the increased availability of amino groups capable of forming hydrogen bonds and interacting with the LAP phase, thereby enhancing the effective connectivity of the polymer–clay network. It is important to note that the swelling results indicate that the influence of DD is not independent of the processing method. For the 1FD samples, the CS4.3 composite with higher DD exhibits a higher swelling ratio than CS1.3, while this relationship is reversed after the second freeze-drying step. This observation indicates that DD affects not only the density of intermolecular interactions but also the structural organization established during processing. On the other hand, reducing the CS concentration from 3 wt.% to 2 wt.% (CS3.2-LAP0.2.1FD, CS3.2-LAP0.1.1FD) leads to a significant decrease in stiffness (6.77 and 5.96 kPa, respectively), while compressive stress remains relatively similar (643.6 and 631.47 kPa) (Figure 7, Table S3), suggesting that LAP still contributes to load-bearing even in less-dense networks. These results are well correlated with the swelling data where a decrease in CS concentration from 3 wt.% to 2% wt.% led to higher equilibrium swelling coefficients, indicating a less compact network with a greater capacity to absorb the solvent. Thus, the decrease in modulus at a lower CS concentration can be attributed not only to a smaller amount of polymer but also to the formation of a network with less-dense cross-links.

A similar but less pronounced behavior is observed for the “2” series, as shown in Figure 6B and summarized in Figure 7 and Table S4. The increase in molar mass from 147 to 305 kDa (CS1.3-LAP0.15.2FD and CS2.3-LAP0.15.2FD) results in only a minor change in stiffness (4.35 to 4.57 kPa) and a moderate increase in compressive stress (472.45 to 503.43 kPa), suggesting a limited contribution from chain entanglement alone. A more pronounced improvement is observed for CS3.3-LAP0.15.2FD and CS4.3-LAP0.15.2FD, where modulus increases to 7.53 kPa and 10.45 kPa (Figure 7 and Table S4), respectively, accompanied by higher stresses (510.03 and 529.9 kPa), highlighting the combined role of molecular weight and DD. As in the “1” series, CS4.3-LAP0.15.2FD (CS with 94% DD) exhibits the best mechanical performance within this group.

The influence of polymer concentration can be assessed by comparing samples containing CS with identical molecular characteristics (256 kDa, DD = 86%). Increasing CS concentration from 1 wt.% (CS3.1-LAP0.1.2FD) to 2 wt.% (CS3.2-LAP0.1.2FD) and 3 wt.% (CS3.3-LAP0.15.2FD) results in a marked increase in compressive modulus from 1.57 to 2.82 and 7.53 kPa, respectively. The higher polymer content likely increases chain entanglement density and the number of polymer–clay interactions, producing a denser network structure and greater resistance to deformation. Interestingly, CS3.1-LAP0.1.2FD exhibits the highest compressive stress (833.7 kPa) despite having the lowest modulus. This behavior indicates that the more flexible network can undergo substantial deformation before failure, allowing efficient dissipation of mechanical energy under compression.

The role of inorganic content can be evaluated by comparing CS3.2-LAP0.2.1FD (10:1) and CS3.2-LAP0.1.1FD (20:1), which have identical CS characteristics and concentration (2 wt.%). Increasing the CS:LAP ratio from 10:1 to 20:1 leads to an increase in compressive stress from 492.9 to 532.6 kPa. This trend was also observed previously for nanocomposite scaffolds based on CS/clay prepared by freeze-drying process [60].

The differences between the 1FD and 2FD materials further highlight the importance of processing-induced network architecture. The swelling data indicate differences in equilibrium swelling between the two preparation strategies, which have been attributed to network compactness variations associated with the rate of CS deprotonation relative to freezing and lyophilization. The pore size data also show that the 2FD procedure generally produces a narrower pore size distribution for several of the formulations with higher CS content. These observations indicate that the freeze-drying strategy indirectly affects mechanical properties by determining the spatial arrangement of polymer chains and LAP domains and, consequently, the size, distribution, and connectivity of the pores [12,38,60]. Therefore, the process history is an important structural variable that should be considered alongside composition when the mechanical response is analyzed. Overall, both series demonstrate that the DD and polymer concentration strongly influence compressive behavior. Higher DD generally increases stiffness, while reductions in polymer concentration primarily decrease the elastic modulus. Although the “1” samples generally exhibit higher compressive modulus values (6–21 kPa vs. 2–10 kPa), compressive stress (627–717 kPa vs. 472–833 kPa) does not follow the same trend across all formulations, as evidenced by the exceptionally high stress sustained by CS3.1-LAP0.1.2FD hydrogels. These results highlight the complex interplay between molecular characteristics, polymer concentration, filler content, and freeze-drying strategy/history in determining the mechanical performance of CS–LAP nanocomposite hydrogels.

### 2.2. Loading and Release of 5-FU in/from CS-LAP Nanocomposite Sponges

The pH-responsiveness of CS-based composite porous materials makes them suitable for oral administration of the drug release systems controlled through a pH-dependent mechanism [12,61,62]. In this work, the loading and release of 5-FU in/from CS-LAP nanocomposite sponges were associated with the preparation conditions. Such CS-LAP nanocomposite sponges have never been evaluated as potential delivery systems for controlled release of 5-FU. The percentages of the drug loaded into some double-component CS-LAP composites are presented in Table 2.

To demonstrate the presence of 5-FU molecules into the CS-LAP composites after the drug loading, FTIR spectra presented in Figure S6 are examined comparative with the FTIR spectrum of 5-FU. Comparing the FTIR spectrum of the 5-FU-loaded CS=LAP nanocomposites with that of the pristine CS-LAP, some additional absorption bands located at 752 cm^−1^, 810 cm^−1^ and 1250 cm^−1^ can be observed, attributed to the out-of-plane bending vibration of -CF-CH- groups and to C-F stretch band associated with the presence of 5-FU [45,48]. These bands can be seen in the FTIR spectrum of 5-FU at 752, 810 and 1246 cm^−1^ and confirm the successful loading of 5-FU within the selected CS-LAP nanocomposite sponges.

**Figure 8:**
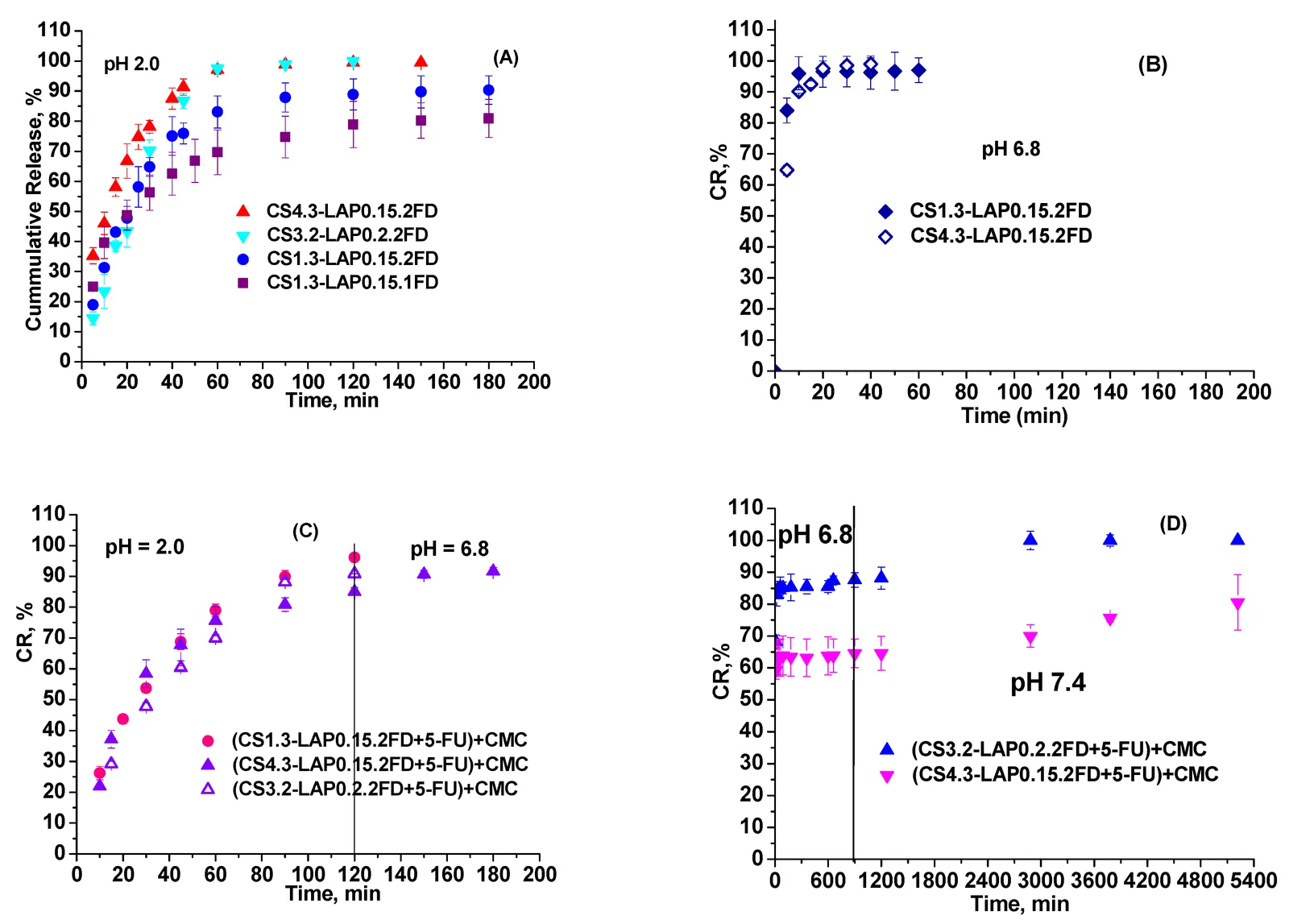
Release kinetics of 5-FU from CS-LAP nanocomposite sponges at pH 2.0 (**A**) and pH 6.8 (**B**); release profiles of 5-FU from the [(CS-LAP)+5-FU]CMC as a function of pH (**C**,**D**).

To gain information on the influence of DD of CS on the drug release, two composite samples differing by the DD of CS used in their construction (CS1.3-LAP0.15.2FD and CS4.3-LAP0.15.2FD, Table 1) were tested for their performances in the release of 5-FU. Information about the influence of the strategy used to fabricate the CS-LAP sponges on the drug release kinetics were received from the comparison between two composite different only by the synthesis strategy (CS1.3-LAP0.15.1FD and CS1.3-LAP0.15.2FD). The release kinetics of 5-FU from the CS-LAP nanocomposites presented in Table 2, at pH 2.0, are depicted in Figure 8A. As can be observed, there is a clear influence of the DD of CS on the release kinetics of 5-FU, as the fastest release occurred from the composite constructed with CS4 (DD = 94%, Table 1). Almost the same profile was identified in the case of the composite constructed with the CS3 at a concentration of 2 wt.% CS. The release rate of 5-FU from the four nanocomposite sponges can be ordered as follows: CS1.3-LAP0.15.1FD < CS1.3-LAP0.15.2FD < CS3.2-LAP0.2.2FD ≤ CS4.3-LAP0.15.2FD. For these composites it must be specified that a common feature was that they completely disintegrated [63,64] in about three hours. This order of drug release would indicate that: (i) the slower release of the drug from the CS1.3-LAP0.15.1FD could be associated with the lower swelling rate of this composite sponge; (ii) the fast drug release observed in the case of CS3.2-LAP0.2.2FD can be attributed to the very high swelling rate (~36, Figure 4B), associated with the CS concentration of 2 wt.%, while the fast release of 5-FU from CS4.3-LAP0.15.2FD is probably related to a lower efficiency of cross-linking at the same CS:LAP ratio. Such composite systems could be recommended for the therapy of gastric cancer, as the drug release of 5-FU in stomach is lately of strong interest [17,50,65].

Completely different release profiles were observed at pH 6.8, when the composites with the fastest release rate of 5-FU, at pH 2.0, were compared (Figure 8B). In this environment, the payload was completely released in about 20 min, without the disintegration of the carrier. To retard the release rate of 5-FU from CS-LAP composite sponges and to better protect the drug stability along the GIT, the drug was sealed inside the porous composites by the complexation of free positive charges of CS with CMC as polyanion, after the drug loading.

The release kinetics of 5-FU at pH 2.0 from (CS1.3-LAP0.15.2FD+5-FU)+CMC, (CS4.3-LAP0.15.2FD+5-FU)+CMC and (CS3.2-LAP0.2.2FD+5-FU)+CMC composites are presented in Figure 8C. It is obvious that the presence of CMC has a positive effect on the drug release, with the slowest release rate found in the case of the composite synthesized with CS4, i.e., with the highest DD [(CS4.3-LAP0.15.2FD+5-FU)+CMC], and the fastest from the composite fabricated with CS1, i.e., with the lowest DD (CS1.3-LAP0.15.2FD+5-FU)+CMC, with (CS3.2-LAP0.2.2FD+5-FU)+CMC located in between. The explanation is connected with the efficiency of the complex formation between CS and CMC, with a stronger interaction and a higher stability being expected in the case of CS4 than in the case of CS1. This assumption is supported by the optical images presented in Figure S7, which show that, during the drug release, the composite (CS1.3-LAP0.15.2FD)+CMC swelled at a much higher level that the other two composites. The release of 5-FU was extended at pH 6.8, in the case of (CS4.3-LAP0.15.2FD+5-FU)+CMC with the release of ~92% of 5-FU during 180 min.

To gain further knowledge about the potential of the CS-LAP sponges as carriers of 5-FU in the GIT environments, two of the above tested composites [(CS4.3-LAP0.15.2FD+5-FU)+CMC and (CS3.2-LAP0.2.2FD+5-FU)+CMC, Figure 8C)] were employed in the drug delivery first at pH 6.8 and then at pH 7.4 (Figure 8D). It is obvious that both composites display a burst release at pH 6.8 followed by a steady release up to 900 min, at a value of CR of about 63 ± 5% for [(CS4.3-LAP0.15.2FD+5-FU)+CMC] and 85.5 ± 2% for [(CS3.2-LAP0.2.2FD+5-FU)+CMC]. After the replacement of the release medium with pH 7.4, a different profile of the drug release was found, consisting of the steady release of drug from the composite [(CS4.3-LAP0.15.2FD+5-FU)+CMC, and a fast release from the composite [(CS3.2-LAP0.2.2FD+5-FU)+CMC] up to 100%. These results support the potential of the [(CS4.3-LAP0.15.2FD+5-FU)+CMC composite to perform as a drug carrier for 5-FU along the GIT environments.

Information about the release mechanism of 5-FU from the CS-LAP and (CS-LAP+5-FU)+CMC nanocomposites, at pH 2.0, were obtained by fitting three kinetic models on the release data plotted in Figure 8A and Figure 8C, respectively. The kinetic models fitted in Figure S8 are first-order kinetics (Equation (1)) [66], the Higuchi model (Equation (2)) [67], and the Korsmeyer–Peppas model (Equation (3)) [68], the equations of which are presented below:

(1) *M_t_* = *M*_0_ − *exp*^−^*^k^*^1∙^*^t^*

(2) *M_t_* = *k_H_t*^1/2^

(3) $$\frac{M_{t}}{M_{\infty }\;}\;=\;k_{KP\;}t^{nr}$$

where *k*_1_ the constant for first-order model; *k_H_* is the Higuchi constant; *M_o_* is the initial amount of drug; *M_t_* and *M_∞_* are the cumulative amounts of drug released at time *t* and the maximum released amount (released at infinite time); *k_KP_* is a constant related to the matrix; and *n_r_* is diffusional exponent which gives indication about the release mechanism.

The kinetic parameters and the values of the coefficient of determination, *R*^2^, for the drug release at pH 2.0, from the CS-LAP composites displayed in Figure 8A, with the linear fit in Figure S8A–C, are presented in Table 3.

The kinetic parameters and the values of the coefficient of determination, *R*^2^, for Higuchi and Korsmeyer–Peppas models, obtained by the linear fit of the drug release data at pH 2.0 (Figure S8B,D), from the [(CS-LAP)+5-FU]+CMC nanocomposites displayed in Figure 8C, are presented in Table 4.

The data presented in Table 3 and Table 4 demonstrate that the coefficients of determination are high for all kinetic models fitted on the experimental data. The discussion of the possible release mechanism was performed based on the Korsmeyer–Peppas kinetic model, which gives the values of index “n”. A Fickian mechanism is indicated by the values of n ≤ 0.45, while values of 0.45 < n < 0.85 indicate that the drug release occurs according to an anomalous mechanism. As can be seen, the data in Table 3 and Table 4 support a Fickian release of 5-FU in the case of the nanocomposites CS1.3-LAP0.15.1FD and CS4.3-LAP0.15.2FD, while the release of 5-FU from the other composites follows an anomalous mechanism.

Some composite formulations based on CS and LAP recently reported in the literature and tested as potential DDSs for ofloxacin [29], DOX [31,32], CCM [34,35] are presented in Table S5. Composite DDSs tested in the controlled release of 5-FU and included in Table S5 are based on IPN(CS+PVA) cross-linked with glutaraldehyde [40], CS nanoparticles ionically stabilized with TPP [41], intercalation of 5-FU molecules in MTT+NaAlg, further stabilized with CS [43], CS microspheres covalently cross-linked with GA [44], bio-nanocomposite hydrogel beads with 5-FU encapsulated in MOF-5 and coated with CMC [48], and NaAlg/Xanthan Gum nanocomposite containing halloysite nanotubes [49]. It is obvious that the systems effective in the controlled release of the low-molar-mass drug 5-FU are stabilized either by covalent cross-linking [40,44] or by further ionic interactions [41,43,45,47,48,49] to protect the drug within the GIT environment and to ensure a slower release of drug.

## 3. Conclusions

CS-LAP nanocomposite sponges with morphology and swelling properties modulated by the DD of CS, CS concentration and the strategy employed to fabricate porous nanocomposites, with potential for oral administration of 5-FU, were successfully achieved in this work. As a function of the swelling capacity, the CS-LAP nanocomposite sponges proved to be suitable for delivery of 5-FU, either in the stomach (pH 2.0) by the release of drug simultaneous with dissolution/disintegration of the composite carrier, or into the small intestine (pH 6.8) and colon (pH 7.4) after the temporary sealing of the drug into the CS-LAP by complexation of CS with CMC. The 5-FU release profiles from CS-LAP nanocomposites clearly demonstrated the influence of synthesis conditions and pH-dependent release behavior, highlighting the potential of these nanocomposites for localized drug delivery to tumors. The results indicate that the CS-LAP/5-FU/CMC nanocomposite could be considered a promising DDS for the oral administration of 5-FU. Nevertheless, further optimization and validation studies are needed to fully assess the potential of these systems, including comprehensive in vitro and in vivo investigations. The additional controls, including free 5-FU, drug-loaded CS–LAP without CMC, drug-loaded CS without LAP, CMC/5-FU without the CS–LAP carrier, and blank carriers, would provide valuable information for further elucidating the specific contribution of each component. These additional control formulations will be included in our future work, together with further in vitro biological assessments, long-term stability and degradation studies, in vivo evaluations, and, ultimately, clinical studies.

It is the future scope of the study the evaluation of the cytotoxicity, biodegradation, biocompatibility, and in vitro/in vivo therapeutic performance of the developed nanocomposites.

## 4. Materials and Methods

### 4.1. Materials

Four CS samples differing by molar mass and DD were purchased from Sigma Aldrich (Burlington, MA, USA) and used as received. The intrinsic viscosity of each sample, dissolved in a 1:1 (*v*/*v*) mixture of 0.3 M CH_3_COOH and 0.2 M CH_3_COONa, was determined with an Ubbelohde viscometer at 25 ± 0.1 °C. The viscometric average molar masses (M_v_), evaluated according to [69], are presented in Table 1. The DD was determined from the liquid state proton spectrum, according to the procedure described in the literature [70]. Laponite RD, with 1000 kg/m^3^ bulk density, surface area of 370 m^2^/g, a chemical composition of SiO_2_ 59.5%, MgO 27.5%, Li_2_O 0.8%, Na_2_O 2.8%, and loss on ignition 8.2%, was obtained from Rockwood Additive Ltd. (BYK-Chemie GmbH, Wesel, Germany). Glacial acetic acid, NaOH p.a. (≥98%), and ethanol p.a., purchased from Chemical Company, were all used as received.

### 4.2. Methods

#### 4.2.1. Preparation of the CS-LAP Porous Nanocomposites

In this work, the as-prepared nanocomposite sponges are denoted as CSx.y-LAPz (Table 1), where CS and LAP indicate chitosan and Laponite RD, respectively, x represents the code of CS sample (1, 2, 3, 4, Table 1), and y and z represent the corresponding concentrations as CS/100 mL H_2_O (*w*/*v*), and LAP/100 mL H_2_O (*w*/*v*), respectively. For example, CS1.3-LAP0.15 (Table 1) means the nanocomposite based on CS1 (M_v_ = 147 kDa and DD = 82%) containing 3% *w*/*v* CS and 0.15% *w*/*v* Laponite RD. After the mild dispersion of the LAP clay in H_2_O to reach the concentrations specified in Table 1, followed by ~2 h of magnetic stirring at room temperature (RT), at a mixing rate of ~100 rpm, the CS powder was dispersed under continuously magnetic mixing and kept under stirring for 30 min, and then glacial acetic acid corresponding to a concentration of 2% (*v*/*v*) was added, with the stirring being kept at the same mixing rate, until a homogeneous mixture was obtained (about 5 h). The homogeneous dispersion of LAP in the CS solution was kept overnight at RT for removal of air bubbles. The CS-LAP mixtures were loaded in syringes of 5 mL, and then frozen overnight in a cryostat at −20 °C. The samples were completely frozen in about 30 min. The frozen monoliths were carefully pushed out from syringes after ~1 min at RT, cut in fragments of about 10 mm, and divided in two portions: the first one was immediately transferred into NaOH 3 M aqueous solution for 24 h, a time sufficient for deprotonation of CS, and the other one into a Freeze Dryer Biobase BK-FD10S device (Jinan, China), for freeze-drying (48 h, at −57 °C and 0.045 mbar). The dried cryostructured CS-LAP sponges were transferred in 3 M NaOH aqueous solution for 24 h, to remove the acetic acid and to stabilize their morphology. Thereafter, the deprotonated CS-LAP sponges were washed with copious amounts of water to neutral pH, frozen overnight at −20 °C, and again lyophilized (2FD) to obtain resilient physically cross-linked CS-LAP nanocomposite sponges. For the fabrication of CS-LAP nanocomposites in this work, the CS4.3-LAP0.15 is given as an example. A weighed amount of 0.045 g LAP was mildly dispersed in 30 mL H_2_O (~2 h) at RT, and after that 0.9 g of CS4 was dispersed and stirred for about 30 min. After that, 0.45 mL acetic acid were dropped and the mixture was kept under stirring for about 5 h. The homogeneous mixture remained overnight at RT and then transferred into six syringes of 5 mL, which were placed into the cryostat at −20 °C, overnight. The syringes were taken out from cryostat one by one, the monoliths were carefully ejected, cut into ~10 mm fragments, the content of three syringes was put in NaOH 3 M and the other fragments were returned into the cryostat for the next step, which was freeze-drying.

#### 4.2.2. Characterization Methods

To evaluate the yield in nanocomposite as a function of fabrication conditions, the samples were further dried under vacuum in the presence of P_2_O_5_, until the constant weight was reached.

The yield was evaluated by Equation (4):

(4) *Y* = (*W*_*d*_/*W*_*m*_) × 100 (%)

where *W_d_*—weight of dried composite; *W_m_*—total weight of initial components.

For the characterization by FTIR spectroscopy, the samples were frozen in liquid nitrogen (LN) and grounded into fine particles using a mortar. FTIR spectra were recorded using a Bruker Vertex FT-IR spectrometer (Bruker, Ettlingen, Germany), with a resolution of 2 cm^−1^ over the range of 4000–400 cm^−1^, through the KBr pellet technique, with about 5 mg of sample in each pellet.

The evaluation of surface morphology for the obtained hydrogels was performed with the help of a Verios G4 UC Scanning electron microscope (Thermo Scientific, Brno, Czech Republic). The samples were fixed on aluminum stubs with double-adhesive carbon tape and afterwards coated with a 7 nm platinum layer using a Leica EM ACE200 Sputter coater prior to examination in order to provide electrical conductivity and prevent charge buildup which can occur during exposure to the electron beam. SEM investigations were carried out in High Vacuum mode using a concentric backscatter electron detector (CBS) at 20 kV accelerating voltage and 6.4 nA spot size, with the magnification being indicated on the micrographs. The average diameter of pores for all the investigated hydrogels was evaluated from the SEM images by ImageJ 1.52a software, measuring approximately 30 pores on three images [38,51]. The identification of the chemical elements of the studied materials was performed by using an energy-dispersive X-ray spectroscopy analyzer (EDAX Octane Elite Energy-Dispersive X-ray Spectroscopy analyzer, Ametek, Berwyn, PA, USA) coupled to Verios G4 UC scanning electron microscope. The EDAX spectra were recorded in different areas of the samples to confirm the presence of LAP in hydrogels and also to obtain the map distribution of the elements within the obtained systems.

Swelling kinetics (swelling ratio, *SR*, as a function of time) were evaluated in distilled water by Equation (5).

(5) *SR* = *W_t_*/*W_d_*

where *W_t_* represents the mass of the swollen gel at time t; *W_d_* is the mass of gel in dry state.

The mean of *SR* measurements on three independent samples has been plotted as a function of time, in the case of the swelling kinetics evaluation.

The mechanical behavior of the swollen sponges was characterized at room temperature using a Shimadzu EZ-SX universal testing machine (EZ-LX/EZ-SX Series, Kyoto, Japan). Cylindrical samples, measuring approximately 9–11 mm in diameter and 8–10 mm in height, were compressed under uniaxial loading with a 50 N load cell at a deformation rate of 1 mm min^−1^. To ensure reproducible contact conditions, an initial preload of 0.1 N was applied before each measurement. Compressive stress, strain, and elastic modulus were determined according to previously established procedures for porous composites [12,38].

#### 4.2.3. Loading/Release of 5-FU

The selected CS-LAP sponge samples were loaded with 5-FU by the sorption-solvent evaporation technique [12,23,47,49]. Solutions of 5-FU in distilled water with a concentration of 5 mg/mL were first prepared and added to certain amounts of composites as carriers up to their maximum sorption capacity was reached (indicated by their equilibrium swelling ratio presented in Figure 4). The samples were kept 24 h in closed bottles, at +4 °C, in the dark, for the drug sorption at equilibrium. After that, monolith fragments loaded with 5-FU were quickly washed with distilled water, to remove the drug on the sponge surface, and transferred into clean bottles, frozen and freeze-dried in a Freeze Dryer Biobase BK-FD10S device (48 h, at −57 °C and 0.045 mbar). All solutions containing the drug were collected and the drug amount was evaluated by UV-Vis. The loading of CS-LAP sponges with 5-FU (DL, %) was evaluated with Equation (6), by weighing the dried samples loaded with 5-FU. The real amount of drug sorbed into the composites was evaluated by subtraction of the nonadsorbed drug amount from the initial amount of drug:

(6) $$\mathrm{DL}=\frac{\mathrm{weight}\;\mathrm{of}\;5-\mathrm{FU}\;\mathrm{in}\;\mathrm{CS}-\mathrm{LAP}\;\mathrm{sponges}}{\mathrm{weight}\;\mathrm{of}\;\mathrm{drug}\;\mathrm{loaded}\;\mathrm{CS}-\mathrm{LAP}\;\mathrm{sponges}}\;\times \;100$$

5-Fluorouracil (FU) was entrapped in the hydrogels, as a model therapeutic agent, and the in vitro release profiles of the drug were established at 37 °C at pH 2.0, 6.8 and 7.4.

To decrease the drug release rate, some of the nanocomposites loaded with 5-FU were electrostatically stabilized by adsorption at equilibrium of CMC with molar mass 90 kDa as aqueous solution with a concentration of 2 wt.%. The samples were kept at +4 °C for ~24 h and then the samples were transferred at −18 °C, overnight, and finally lyophilized for 48 h.

Thus, the in vitro release of 5-FU at pH 2.0 and 6.8 was performed by immersing the sample loaded with 5-FU in 10 mL release medium. At predetermined time intervals, 1 mL of supernatant was withdrawn and analyzed for the concentration of 5-FU at *λ_max_* of 266 nm using the UV-Vis calibration curve [71] previously recorded with a spectrophotometer (SPECORD 200 Analytik Jena) (Figure S9). The removed solution was replaced with an identical volume of fresh releasing solution. When two environments with different pH were explored (pH 2.0 followed by pH 6.8, or pH 6.8 followed by pH 7.4), the first release medium was replaced with 10 mL of the second medium, for a certain release duration. The cumulative release (CR) of 5-FU was calculated with Equation (7):

(7) $$\mathrm{Cummulative}\;\mathrm{release}=\left[\frac{10C_{n}+\;\sum VC_{n-1}}{m_{o}}\right]\times \;100$$

where C_n_ and C_n−1_ are the concentrations of 5-FU (mg L^−1^) in the releasing medium after n and (n − 1) withdrawing steps; n is the number of withdrawing steps of the release medium; V is the volume of sample (1 mL); and m_o_ is the amount of drug loaded in the sample. All the loading and release experiments were done in triplicate.

## Figures and Tables

**Table 1:** Sample codes, compositions and yields.

Sample Code *	Chitosan			Laponite RD, %, *w*/*v*	CS:LAP Ratio	Yield, %	
	M_v_, kDa	DD, %	%, *w*/*v*			1FD	2FD
CS1.3-LAP0.15	147	82	3	0.15	20:1	84.75	87.89
CS2.3-LAP0.15	305	82	3	0.15	20:1	86.83	86.57
CS3.3-LAP0.15	256	86	3	0.15	20:1	89.19	88.84
CS4.3-LAP0.15	207	94	3	0.15	20:1	91.86	89.13
CS3.2-LAP0.1	256	86	2	0.1	20:1	89.25	87.02
CS3.2-LAP0.2	256	86	2	0.2	10:1	88.3	87.81
CS3.1-LAP0.1	256	86	1	0.1	10:1	82.1	81.56

* The codes of CS were CS1, CS2, CS3 and CS4, having the molar mass of 147 kDa, 305 kDa, 256 kDa and 207 kDa.

**Table 2:** Drug loading of CS-LAP composites tested for drug release as a function of pH.

Sample Code *	CS1.3-LAP0.15.1FD	CS1.3-LAP0.15.2FD	CS4.3-LAP0.15.2FD	CS3.2-LAP0.2.2FD
Drug Loading, %	7.482	11.02	9.252	10.76

* The sample code is identical with that in Table 1.

**Table 3:** Kinetic parameters for the release of 5-FU from CS-LAP nanocomposites.

Kinetic Model	Kinetic Parameters	CS-LAP Sponges			
		CS1.3-LAP0.15.1FD	CS1.3-LAP0.15.2FD	CS3.2-LAP0.1-2FD	CS4.3-LAP0.15.2FD
Higuchi	k_H_	6.7676	12.74	17.01	11.72
	R^2^	0.9345	0.9866	0.9776	0.9736
Korsmeyer–Peppas	n_r_	0.3028	0.6294	0.8555	0.446
	k_KP_ (min^−nr^)	0.2466	0.081	0.03632	0.1768
	R^2^	0.9863	0.9853	0.9877	0.9932
First order	k_t_	0.9876	0.9709	0.9532	0.9518
	R^2^	0.9135	0.9872	0.9731	0.9929

**Table 4:** Kinetic parameters for the release of 5-FU from [(CS-LAP)+5-FU]+CMC nanocomposites.

Kinetic Model	Kinetic Parameters	[(CS-LAP)+5-FU]+CMC Nanocomposites		
		CS1.3-LAP.0.15.2FD	CS3.2-LAP0.2.2FD	CS4.3-LAP0.15.2FD
Higuchi	k_H_	11.4	11.45	11.4
	R^2^	0.9545	0.9963	0.9553
Korsmeyer–Peppas	n_r_	0.6124	0.6605	0.6637
	k_KP_ (min^−nr^)	0.0677	0.05364	0.05115
	R^2^	0.9909	0.9876	0.9307

## Data Availability

The data presented in this study are available on request from thecorresponding author.

## Supplementary Materials

The following supporting information can be downloaded at https://www.mdpi.com/article/10.3390/gels12090771/s1, Figure S1. SEM micrographs of CS-LAP sponges: scale bar is 1 µm and mag 20,000× in all micrographs; Figure S2. EDX spectra of CS-LAP cryogels as a function of the CS:LAP sets (according to Table 1); Figure S3. EDX data of O and Na for the nanocomposite sponges CS-LAP: (1) CS1.3-LAP0.15.1FD; (2) CS1.3-LAP0.15.2FD; (3) CS2.3-LAP0.15.1FD; (4) CS2.3-LAP0.15.2FD; (5) CS3.3-LAP0.15.1FD; (6) CS3.3-LAP0.15.2FD; (7) CS4.3-LAP0.15.1FD; (8) CS4.3-LAP0.15.2FD; (9) CS3.2-LAP0.2.1FD; (10) CS3.2-LAP0.2.2FD; (11) CS3.2-LAP0.1.1FD; (12) CS3.2-LAP0.1.2FD; Figure S4. FTIR spectra of chitosan (A), Laponite RD (B), CS-LAP composites prepared with CS2 (C) and CS-LAP composites prepared with CS3 (D); Figure S5. The initial linear regions of the stress–strain curves and the linear fits to the experimental data of CS–LAP nanocomposites used to calculate the compressive moduli: (A) “1FD” series and (B) “2FD” series; Figure S6. (A) FTIR spectra of 5-FU and CS1.3-LAP0.15 loaded with 5-FU; (B) FTIR spectra of CS4.3-LAP0.15 and CS3.2-LAP0.2 loaded with 5-FU; Figure S7. Optical images of the CS-LAP/CMC nanocomposites after the release of 5-FU at pH 2.0; Figure S8. Fitting of kinetic models for the release of 5-FU from CS-LAP nanocomposites (A–C) and from [(CS-LAP)+%-FU] +CMC (D,E); Figure S9. Calibration curve of 5FU in PBS, pH 7.4; Table S1. Main absorption bands in the FTIR spectra of the CS-LAP nanocomposite sponges and their assignment; Table S2. Swelling ratio of the composite samples selected for the tests of 5-FU release at pH 2.0 and 6.8; Table S3. Values of compressive elastic modulus, compressive nominal stress, and strain of CS-LAP nanocomposite sponges obtained by one freeze-drying step (1FD); Table S4. Values of compressive elastic modulus, compressive nominal stress, and strain of CS-LAP nanocomposite sponges obtained by two freeze-drying steps (2FD); Table S5. Comparative table with results regarding DDS constructed with CS and LAP found in the literature. The number of references is identical with that given in the main text.
