# Supplementary material for: Physicochemical and Mechanical Evaluation of Nanocomposite Cryogels Based on Chitosan and Laponite and Their Potential for Drug Delivery
Source: Gels. 2026 Aug 27;12(9):771. doi: 10.3390/gels12090771 (PMC13606705; doi:10.3390/gels12090771)
Supplement: Supplementary file 1 [file gels-12-00771-s001.zip › gels-4484971-supplementary.pdf]

Supplementary Information

Physicochemical and Mechanical Evaluation of Nanocomposite Cryogels Based on Chitosan and Laponite and Their Potential for Drug Delivery

Ecaterina Stela Dragan[1*], Maria Valentina Dinu[1], Maria Marinela Lazar[1] and Daniela Rusu[1]

"Petru Poni" Institute of Macromolecular Chemistry, Department of Functional Polymers; Grigore Ghica Voda Alley 41 A, 700487 Iasi, Romania

* Correspondence: sdragan@icmpp.ro (E.S.D.)

Figure S1. SEM micrographs of CS-LAP sponges: Scale bar is 1 μm and mag 20000x in all micrographs.

Figure S2. EDX spectra of CS-LAP cryogels as a function of the CS : LAP sets (according to Table 1).

Figure S3. EDX data of O and Na for the nanocomposite sponges CS-LAP: (1) CS1.3-LAP0.15.1FD; (2) CS1.3-LAP0.15.2FD; (3) CS2.3-LAP0.15.1FD; (4) CS2.3-LAP0.15.2FD; (5) CS3.3-LAP0.15.1FD; (6) CS3.3-LAP0.15.2FD; (7) CS4.3-LAP0.15.1FD; (8) CS4.3-LAP0.15.2FD; (9) CS3.2-LAP0.2.1FD; (10) CS3.2-LAP0.2.2FD; (11) CS3.2-LAP0.1.1FD; (12) CS3.2-LAP0.1.2FD.

Figure S4. FTIR spectra of chitosan (A), Laponite RD (B), CS-LAP composites prepared with CS2 (C) and CS-LAP composites prepared with CS3 (D).

Figure S5. The initial linear regions of the stress–strain curves and the linear fits to the experimental data of CS–LAP nanocomposites used to calculate the compressive moduli: (A) "1FD" series and (B) "2FD" series.

Figure S6. (A) FTIR spectra of 5-FU and CS1.3-LAP0.15 loaded with 5-FU; (B) FTIR spectra of CS4.3-LAP0.15 and CS3.2-LAP0.2 loaded with 5-FU.

Figure S7. Optical images of the CS-LAP/CMC nanocomposites after the release of 5-FU at pH 2.0.

Figure S8. Fitting of kinetic models for the release of 5-FU from CS-LAP nanocomposites (A–C) and from [(CS-LAP)+%-FU] +CMC (D,E).

Figure S9. Calibration curve of 5FU in PBS, pH 7.4.

Table S1. Main absorption bands in the FTIR spectra of the CS-LAP nanocomposite sponges and their assignment.

Table S2. Swelling ratio of the composite samples selected for the tests of 5-FU release in pH 2.0 and 6.8.

Table S3. Values of compressive elastic modulus, compressive nominal stress, and strain of CS-LAP nanocomposite sponges obtained by one freeze drying step (1FD).

Table S4. Values of compressive elastic modulus, compressive nominal stress, and strain of CS-LAP nanocomposite sponges obtained by two freeze drying steps (2FD).

Table S5. Comparative table with results regarding DDS constructed with CS and LAP found in literature. The number of references is identical with that given in the main text.

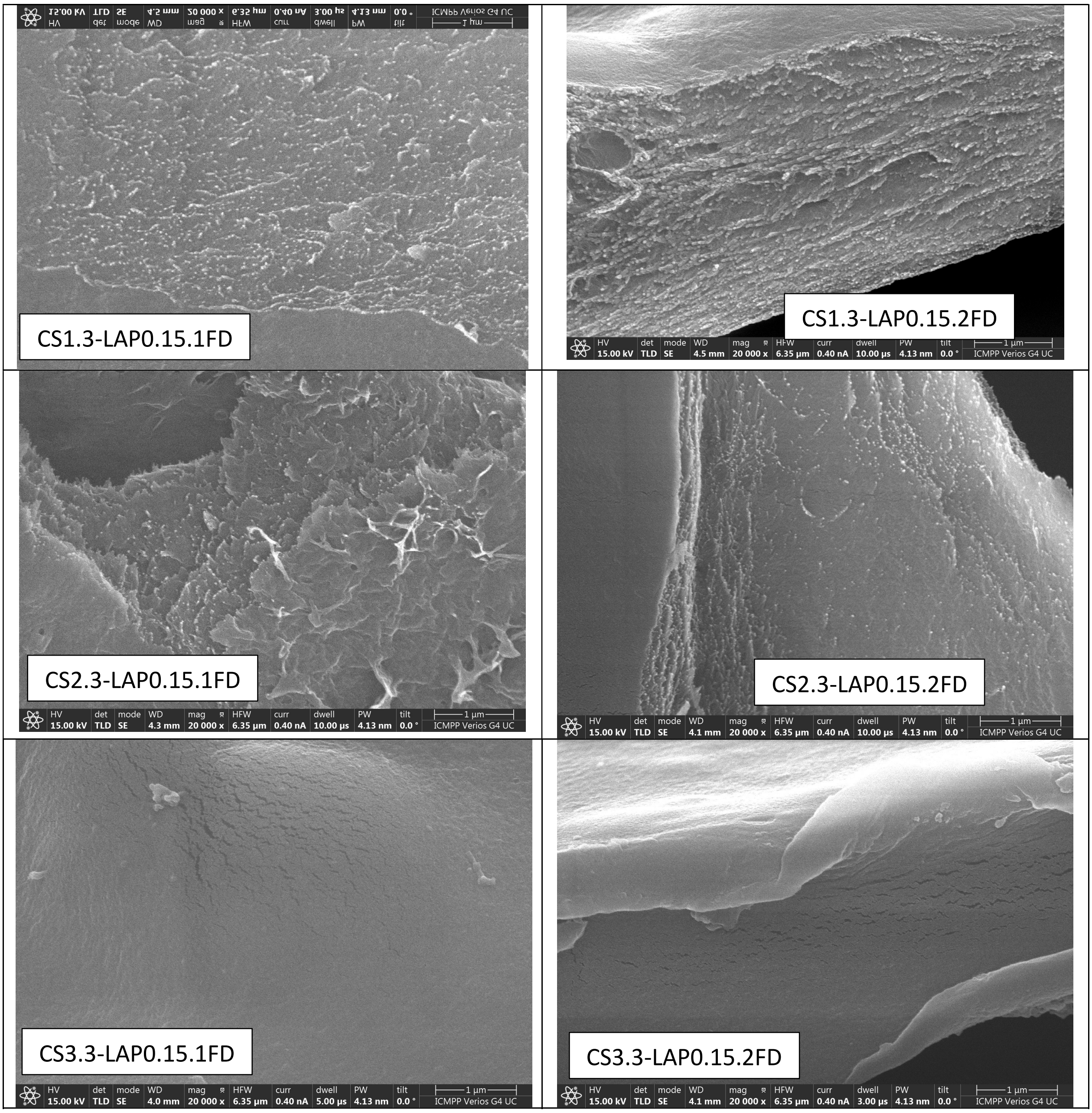

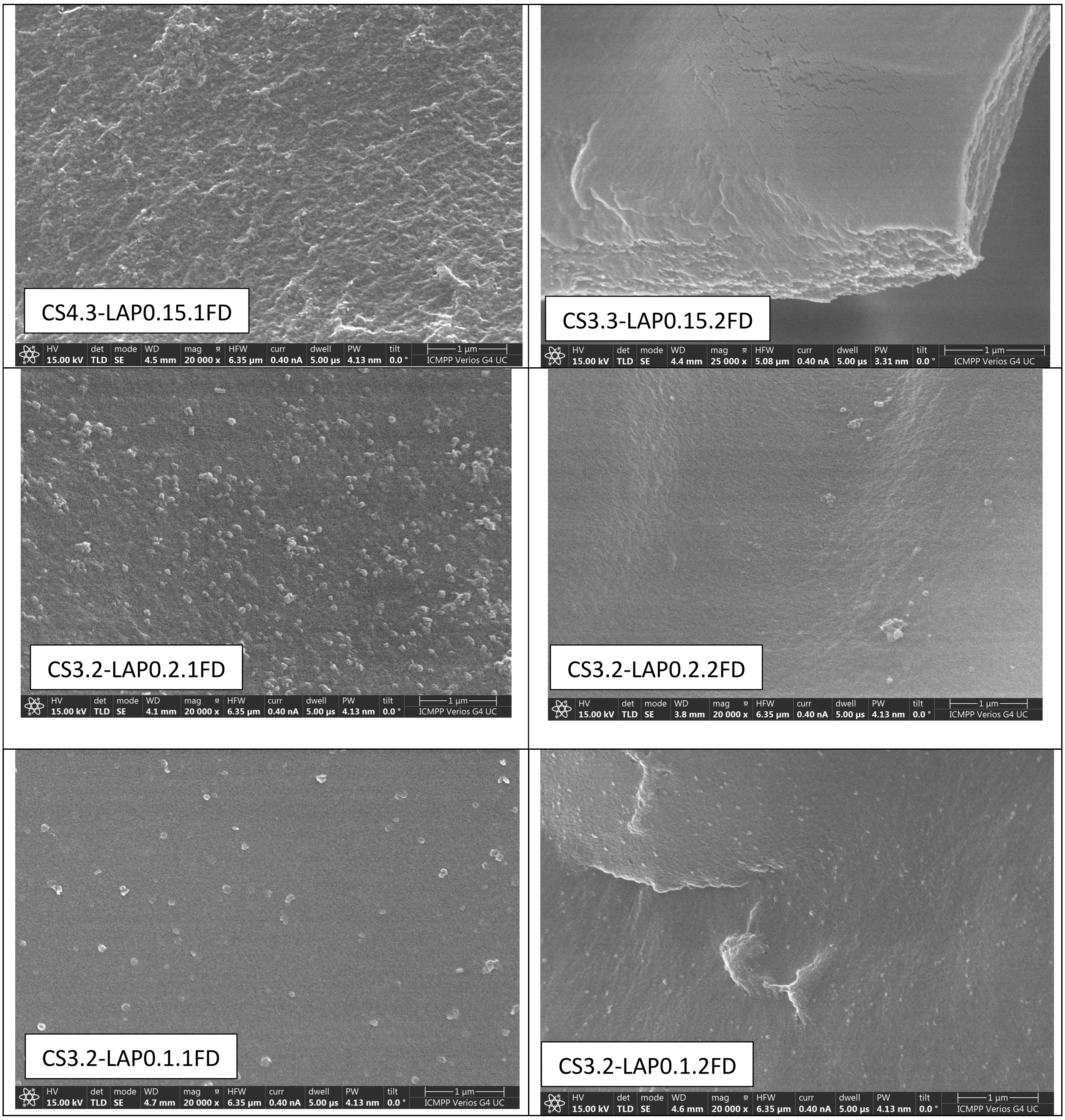

Figure S1. SEM micrographs of CS-LAP sponges: Scale bar is 1 μm and mag 20000x in all micrographs.

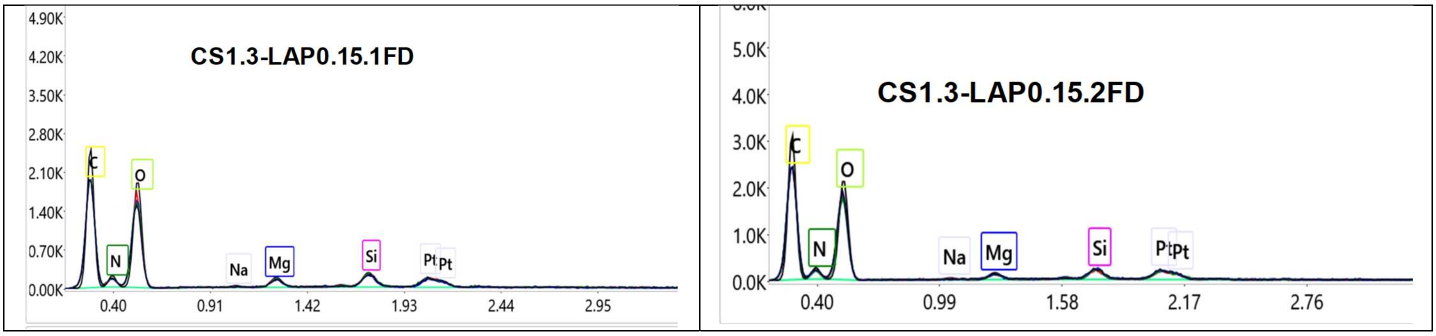

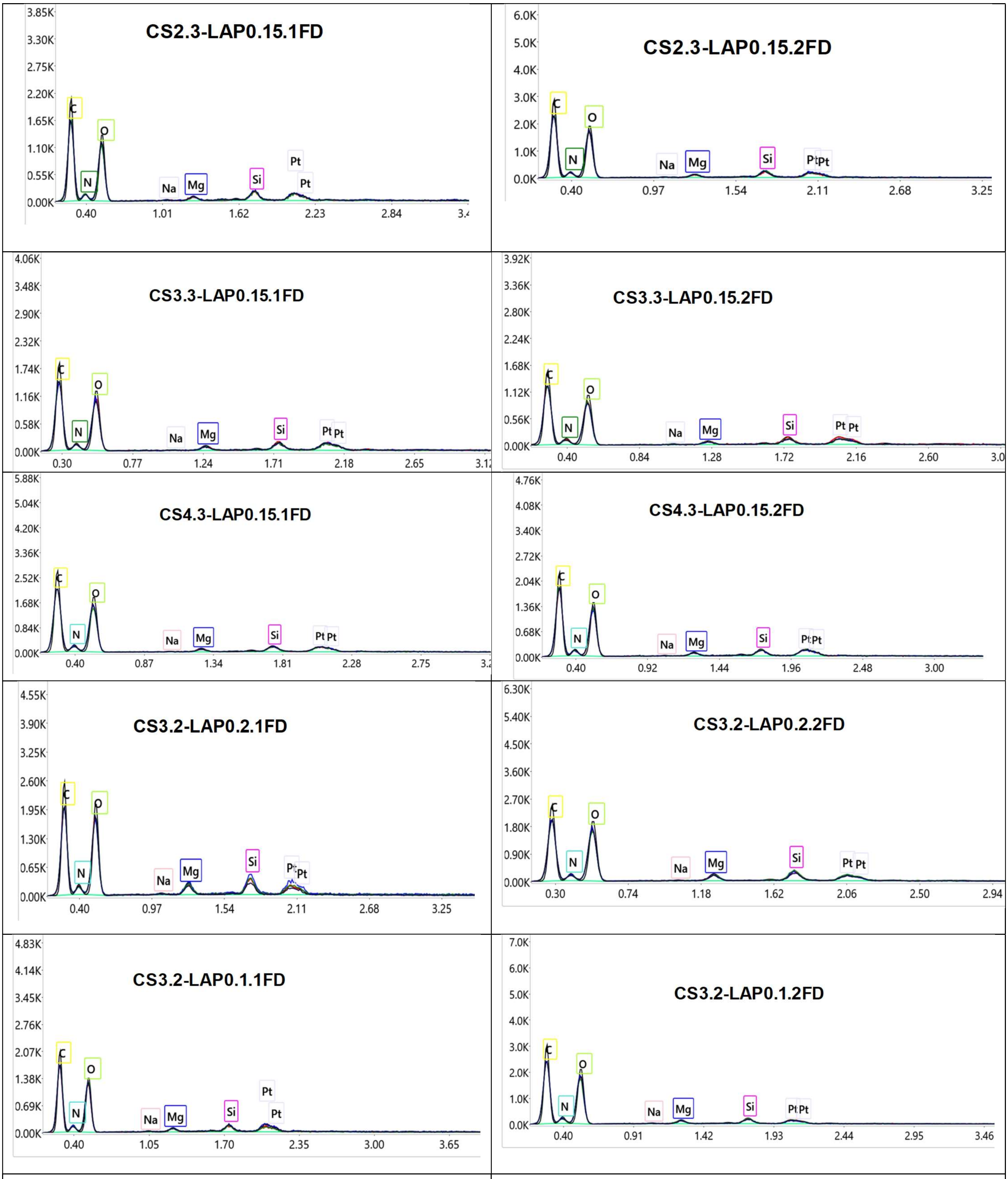

S7a	S7b

Figure S2. EDX spectra of CS-LAP cryogels as a function of the CS : LAP sets (according to Table 1).

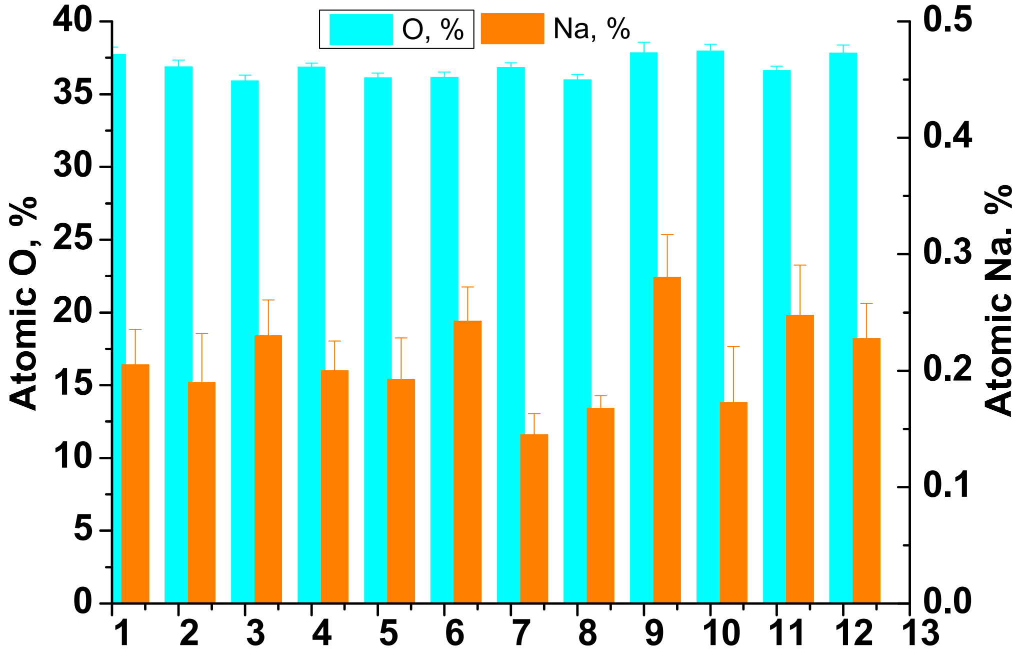

Figure S3. EDX data of O and Na for the nanocomposite sponges CS-LAP: (1) CS1.3-LAP0.15.1FD; (2) CS1.3-LAP0.15.2FD; (3) CS2.3-LAP0.15.1FD; (4) CS2.3-LAP0.15.2FD; (5) CS3.3-LAP0.15.1FD; (6) CS3.3-LAP0.15.2FD; (7) CS4.3-LAP0.15.1FD; (8) CS4.3-LAP0.15.2FD; (9) CS3.2-LAP0.2.1FD; (10) CS3.2-LAP0.2.2FD; (11) CS3.2-LAP0.1.1FD; (12) CS3.2-LAP0.1.2FD

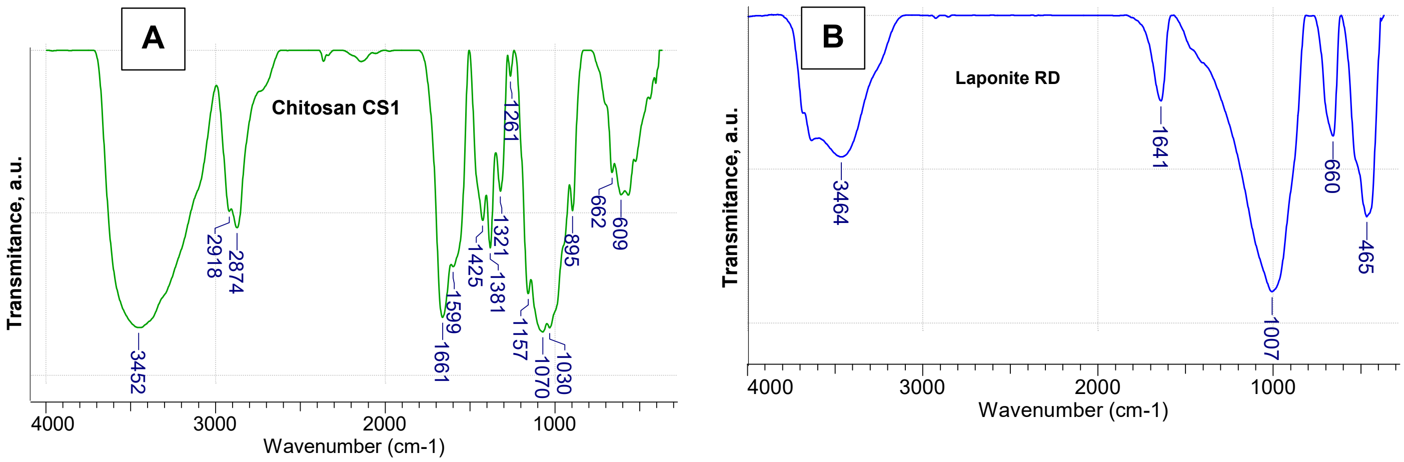

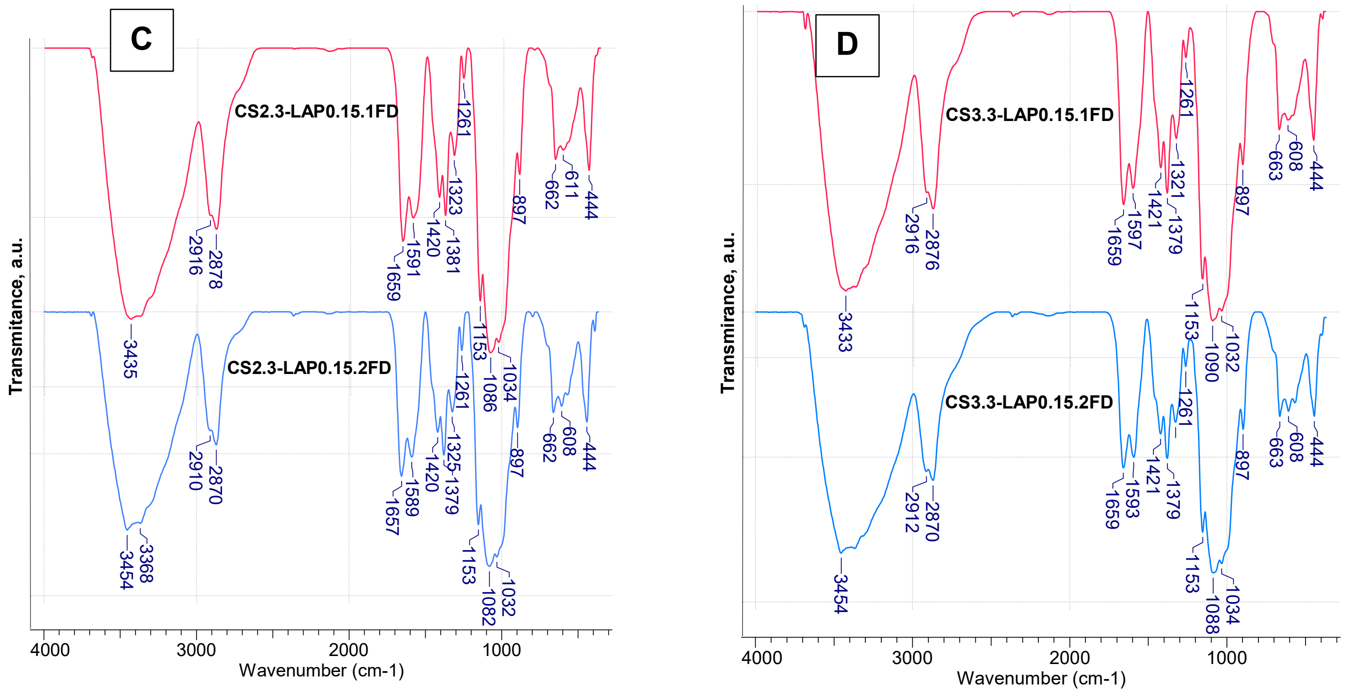

Figure S4. FTIR spectra of chitosan (A), Laponite RD (B), CS-LAP composites prepared with CS2 (C) and CS-LAP composites prepared with CS3 (D).

Table S1. Main absorption bands in the FTIR spectra of the CS-LAP nanocomposite sponges and their assignment.*

Sample Code/wavenumber, cm^{-1}	Assignment							
	C-H stretch in CH_2 groups	C=O stretching vibration of acetamide groups (amide I)	NH in primary amino groups	symmetrical deformation mode of CH_3	C-N in amide III	Anti-symmetric stretching of C-O in C-O-C bridge in CS	skeletal vibration involving C-O stretching	stretching vibration of Si-O-Mg bonds
CS1.3-LAP0.15.1FD Figure 5A	2916; 2878	1659	1566	1379	1321	1153; 1084	1034	444
CS1.3-LAP0.15.2FD Figure 5A	2916; 2876	1659	1591	1379	1321	1153; 1080	1034	444
CS2.3-LAP0.15.1FD Figure S4C	2916; 2878	1659	1591	1381	1323	1153; 1086	1034	444
CS2.3-LAP0.15.2FD Figure S4C	2910; 2870	1659	1589	1379	1325	1153; 1090	1032	444
CS3.3-LAP0.15.1FD Figure S4D	2916; 2876	1659	1597	1379	1321	1153; 1090	1032	444
CS3.3-LAP0.15.2FD Figure S4D	2912; 2870	1659	1593	1379	1321	1153; 1088	1034	444

CS4.3-LAP0.15.1FD Figure 5B	2918; 2876	1653	1597	1380	1325	1153; 1086	1038	444
CS4.3-LAP0.15.2FD Figure 5B	2910; 2870	1653	1591	1381	1331	1153; 1090	1034	444
CS3.2-LAP0.2.1FD Figure 5C	2916; 2876	1659	1599	1381	1325	1153; 1088,	1038	444
CS3.2-LAP0.2.2FD Figure 5C	2914; 2872	1659	1595	1381	1331	1153; 1084	1034	444
CS3.2-LAP0.1.1FD Figure 5D	2916; 2876	1659	1599	1379	1325	1153; 1080	1036	444
CS3.2-LAP0.1.2FD Figure 5D	2912; 2872	1659	1593	1379	1331	1153; 1080	1034	444

- All FTIR spectra display an intense band located at 1261 cm-1 assigned to the C-N stretching modes.

Table S2. Swelling ratio of CS-LAP nanocomposites selected for the experiments of 5-FU release.

Sample Code*	Swelling ratio (SR)	
	pH 2	pH 6.8
CS1.3-LAP0.15.1FD	3.224 ± 0.105	3.347 ± 0.04
CS1.3-LAP0.15.2FD	5.570 ± 0.486	8.878 ± 0.345
CS4.3-LAP0.15.2FD	9.641 ± 0.949	8.889 ± 0.223
CS3.2-LAP0.2 .2FD	16.701 ± 0.704	11.274 ± 2.372

- Sample code is identic with that in Table 1.

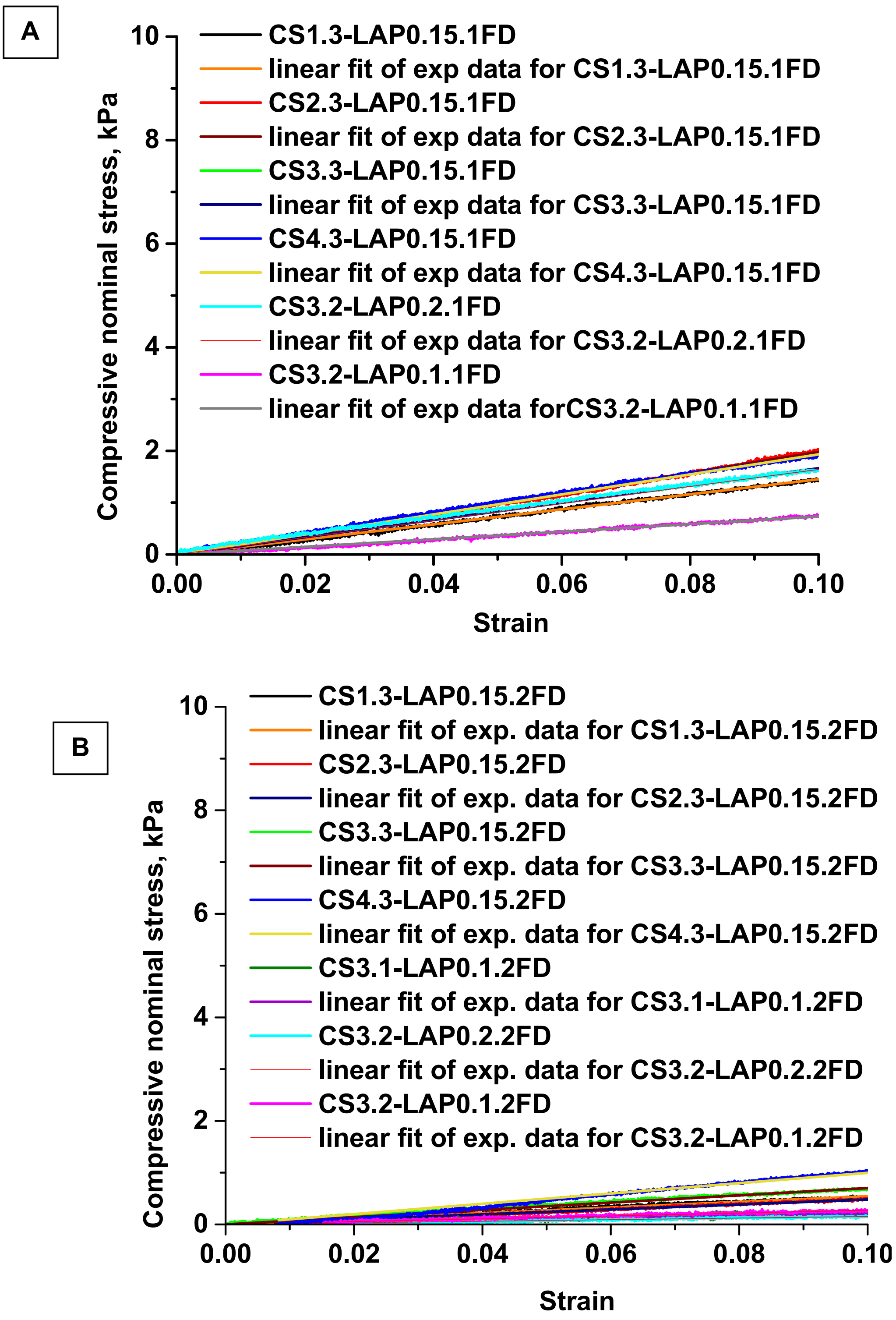

Figure S5. The initial linear regions of the stress–strain curves and the linear fits to the experimental data of CS–LAP nanocomposites used to calculate the compressive moduli: (A) “1FD” series and (B) “2FD” series.

Table S3. Values of compressive elastic modulus, compressive nominal stress, and strain of CS-LAP nanocomposite sponges obtained by one freeze drying step (1FD).

Sample code	Compressive elastic modulus, kPa	R^2	Compressive nominal stress, kPa	Strain%
CS1.3-LAP0.15.1FD	10.82 ±3.68	0.999	658.25± 1.45	88.4± 0.1
CS2.3-LAP0.15.1FD	18.68 ±0.81	0.999	717.37 ±24.78	83.53 ±4.45
CS3.3-LAP0.15.1FD	16.36 ±0.70	0.999	627.00 ±12.90	89.05 ±0.65
CS4.3-LAP0.15.1FD	20.98 ±1.65	0.999	633.07 ±41.61	94.40±0.57
CS3.2-LAP0.2.1FD	6.77 ±2.40	0.998	643.6 ±4.40	84.15 ±5.35
CS3.2-LAP0.1.1FD	5.96 ±0.97	0.997	631.47 ±40.36	86.56 ±5.74

Table S4. Values of compressive elastic modulus, compressive nominal stress, and strain of CS-LAP nanocomposite sponges obtained by two freeze drying steps (2FD).

Sample code	Compressive elastic modulus, kPa	R^2	Compressive nominal stress, kPa	Strain%
CS1.3-LAP0.15.2FD	4.35 ±0.91	0.999	472.45± 6.22	89.37±0.083
CS2.3-LAP0.15.2FD	4.57±1.67	0.999	503.43 ±38.39	92.23 ±0.688
CS3.3-LAP0.15.2FD	7.53 ±0.37	0.999	510.03 ±21.55	87.23 ±2.03
CS4.3-LAP0.15.2FD	10.45 ±0.83	0.999	529.9 ±6.75	87.06±5.15
CS3.1-LAP0.1.2FD	1.57±0.61	0.950	833.7±9.3	76.10±4.9

CS3.2-LAP0.2.2FD	2.19 ±0.46	0.998	492.9 ±24.17	86.67 ±2.80
CS3.2-LAP0.1.2FD	2.82±0.49	0.993	532.6±1.7	89.26±3.07

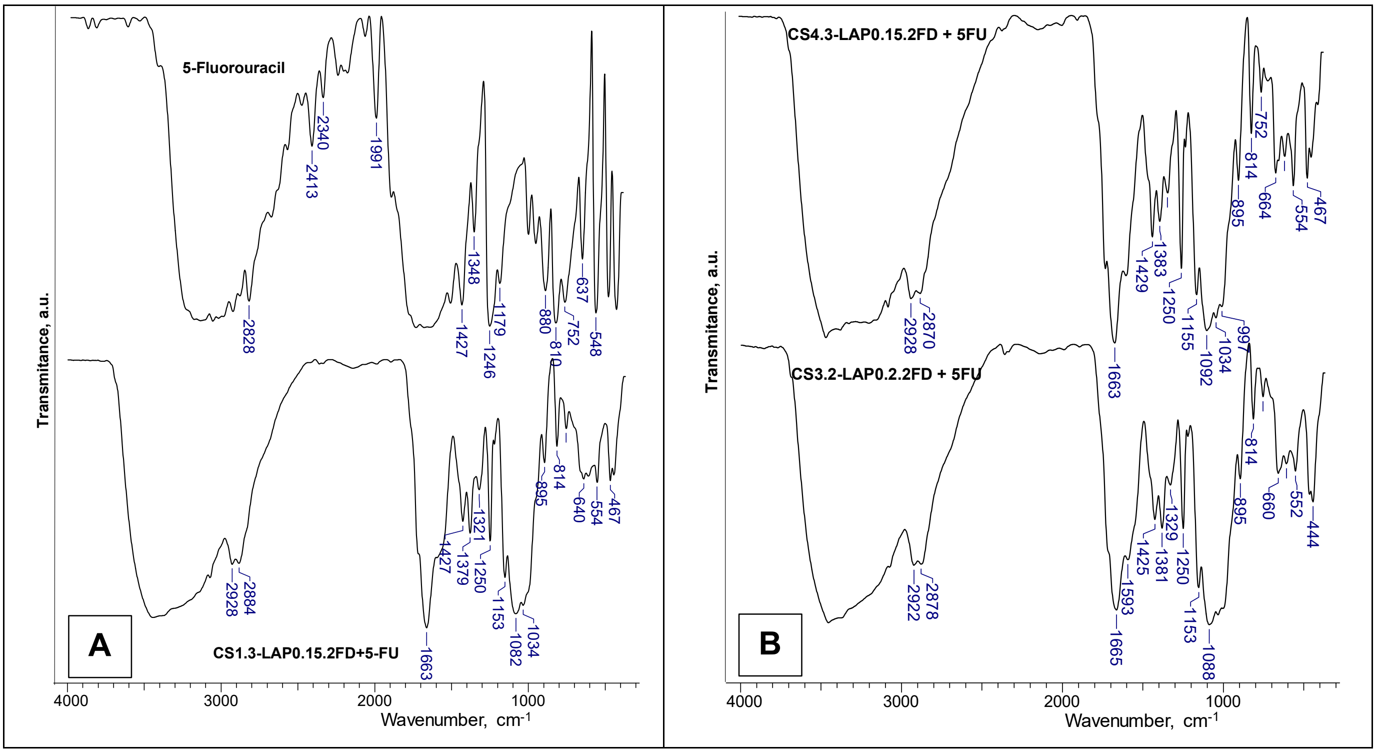

Figure S6. (A) FTIR spectra of 5-FU and CS1.3-LAP0.15 loaded with 5-FU; (B) FTIR spectra of CS4.3-LAP0.15 and CS3.2-LAP0.2 loaded with 5-FU.

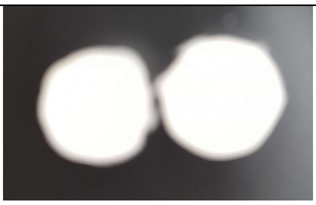 (CS1.3-LAP0.15.2FD)+CMC, loaded with 5-FU	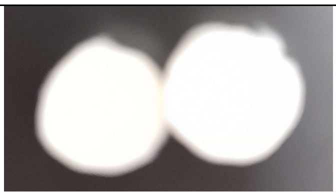 (CS4.3-LAP0.15.2FD)+CMC, loaded with 5-FU	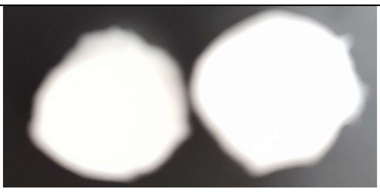 (CS3.2-LAP0.2.2FD) + CMC, loaded with 5-FU
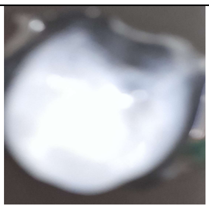 (CS1.3-LAP0.15.2FD + 5-FU) + CMC, after drug release at pH 2.0	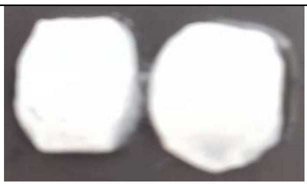 (CS4.3-LAP0.15.2FD + 5-FU)+CMC, after drug release at pH 2.0	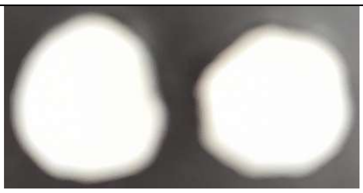 (CS3.2-LAP0.2.2FD + 5-FU) + CMC, after drug release at pH 2.0

Figure S7. Optical images of the CS-LAP/CMC nanocomposites after the release of 5-FU at pH 2.0.

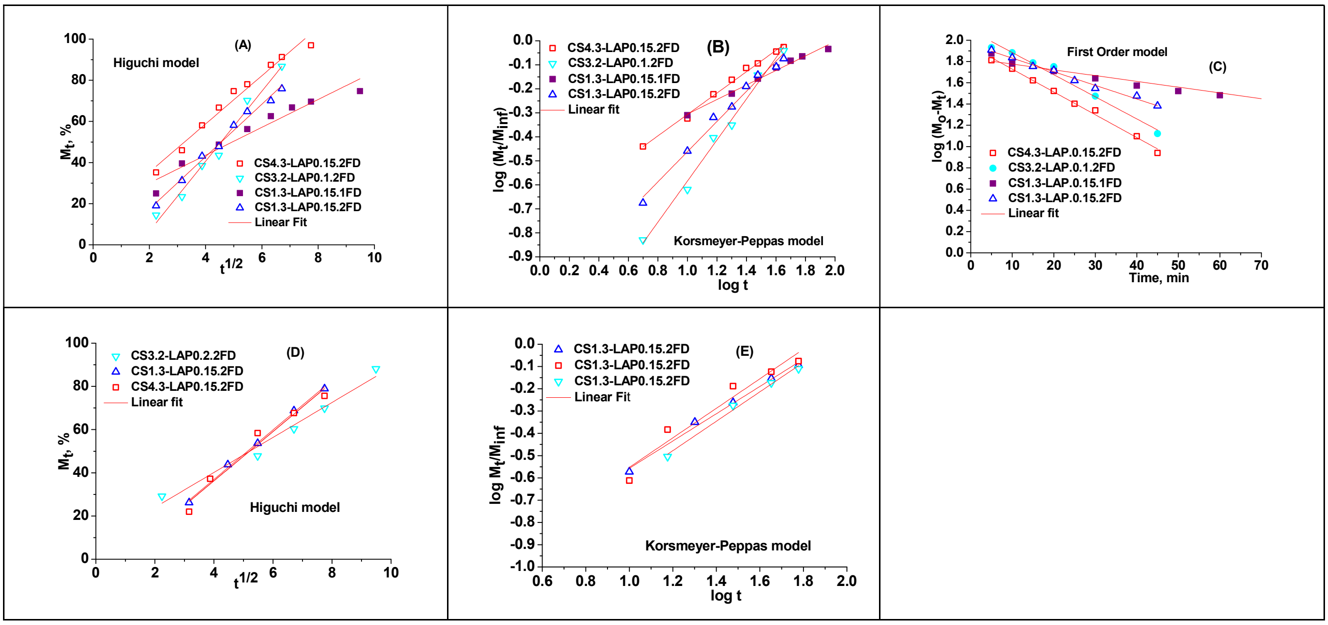

Figure S8. Fitting of kinetic models for the release of 5-FU from CS-LAP nanocomposites (A, B, C) and from [(CS-LAP)+%-FU] +CMC (D, E).

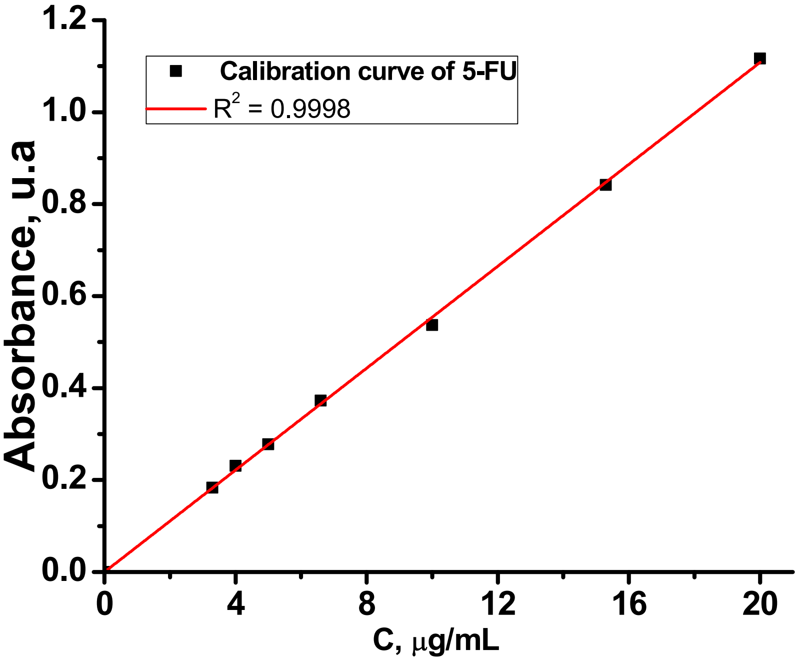

Figure S9. Calibration curve for 5-FU in PBS, pH 7.4.

Table S5. Comparative table with results regarding DDS constructed with CS and LAP found in literature. The number of references is identical with that given in the main text.

Nr.	Drug Delivery System	Drug loading/released	Conditions/disease	Ref.
1	(CS+LAP)+HA; to overcome the burst release of drug	Ofloxacin	pH 7.4; pH 1.2<br>25 °C; 24 h	29
2	CS-LAP nanoparticles in TPP	Doxorubicin and saffron	The effect of cross-linking on the release kinetics was evaluated at pH 5.5 and 7.4	31
3	M(LAP/k-carrageenan)CS core/shell	Doxorubicin	pH: 5.5; 7.4<br>a sustained release of DOX was observed at pH 5.5	32
4	(LAP/CS)PVA core/shell	Curcumin for breast cancer cells	pH: 5.5; 7.4<br>a high release rate at pH 5.5	34
5	(CS/LAP-CCM) nanopart. ionically cross-linked with TPP	Curcumin for chemotherapy	pH: 1.2; 5.5; 7.4	35
6	IPN(CS+PVA) cross-linked with either GA or γ-irradiation; CS with: Mv = 492 kDa and DD = 67.2%	5-FU	pH 2.1; 7.4; 37 °C	40
7	TPP cross-linked CS nanopart; CS with: Mv = 190-310 kDa and DD = 75-85%	5-FU	pH 3; 4; 5; 6; 7.4<br>the highest release rate at pH 5	41
8	Drug molecules intercalated in MTT, + NaAlg, then dropped into CS solution to form complex nanocomposites	5-FU	Colorectal cancer; slow release after burst release, in all cases	43
9	Infl. of CS molar mass and concentr.; microspheres cross-linked with GA	5-FU	pH 6.0 and pH 7.4<br>faster release in pH 6.0	44
10	Drug encapsulated in methyl cellulose/CS microspheres coated or uncoated with NaAlg (core/shell)	5-FU	pH 1.2, 2 h; pH 6.8, 2 h;<br>pH 7.4, 2 h; Colon cancer	45
11	CS/PAA/Fe_3O_4 nanocomposite	5-FU	pH 7.4, 37 °C<br>Colon and rectal cancer	47
12	Bio-nanocomposite hydrogel beads with 5-FU encapsulated in MOF-5 and coated with CMC	5-FU	pH 6.8 + pH 7.4, the highest amount of 5-FU has been	48

			released in a controlled manner; colon cancer	
13	NaAlg/Xanthan Gum nanocomposite containing halloysite nanotubes	5-FU	Cervical cancer therapy	49
